# Sensory Neuroimmunology: Bidirectional Neuro-Immune Circuits Governing Pain, Itch, Inflammation, and Host Defense at Barrier Surfaces

**DOI:** 10.3390/biology15100756

**Published:** 2026-05-09

**Authors:** Reza Mosaddeghi-Heris, Nasrin Forghani, Negin Safari Dehnavi, Maryam Saberivand, Amir Tahavvori, Sohrab Azin, Niloofar Taheri, Paolo Martelletti

**Affiliations:** 1 Neurosciences Research Center (NSRC), Tabriz University of Medical Sciences, Tabriz 5165990001, Iran; rezamosaddeghi1375@gmail.com (R.M.-H.); nasrin.forghani98@gmail.com (N.F.); 2 Sina Trauma and Surgery Research Center, Tehran University of Medical Sciences (TUMS), Tehran 1136746911, Iran; negin.safari8227@gmail.com; 3 Connective Tissue Diseases Research Center, Tabriz University of Medical Sciences, Tabriz 5166614756, Iran; m.saberivand95@gmail.com; 4 Laboratory for Critical Care Physiology, Feinstein Institutes for Medical Research, Northwell Health System, Manhasset, NY 11030, USA; atahavvori@northwell.edu; 5 Student Research Committee, Gonabad University of Medical Sciences, Gonabad 9691833181, Iran; sohrab.azin.75119@gmail.com; 6 Department of Psychiatry and Behavioral Sciences, Stanford University, Palo Alto, CA 94305, USA; 7 School of Health, Unitelma Sapienza University, 00185 Rome, Italy; 8 Tongji Medical College, Huazhong University of Science and Technology, Wuhan 430030, China

**Keywords:** sensory neuroimmunology, neuropeptides, TRP channels, itch, cough, barrier immunity

## Abstract

Sensory neurons at barrier tissues were once seen as passive detectors of environmental stimuli. However, in the last five years, increasing evidence has challenged this view, redefining these cells as active immune sentinels that directly affect tissue immunity in the skin, lungs, and gastrointestinal tract. Nociceptors and pruriceptors express various immune-sensing receptors, including Toll-like receptors, cytokine receptors, and alarmin sensors, which allow them to directly detect pathogens, allergens, and tissue damage. When activated, sensory neurons quickly release neuropeptides such as calcitonin gene-related peptide (CGRP), substance P, vasoactive intestinal peptide (VIP), and PACAP (pituitary adenylate cyclase-activating polypeptide), which guide immune cell recruitment, activation, and resolution. Reciprocally, immune-derived mediators, including IL-33, IL-31, thymic stromal lymphopoietin (TSLP), IL-4/IL-13, and TNF-α, modulate neuronal excitability and plasticity, forming bidirectional neuroimmune circuits that control inflammation, host defense, pain, and itch. Landmark studies published in 2024–2025, including neuronal control of gut Treg function and the identification of sensory nerve immune niches, have further refined this framework and revealed tissue-specific circuit specialization. This review synthesizes recent insights from molecular, cellular, and systems levels into the sensory neuroimmune axis, emphasizes its protective versus pathogenic roles, and critically evaluates emerging therapeutic strategies and safety concerns, positioning sensory neuroimmunology as a unifying framework for tissue barrier homeostasis and disease.

## Simple Summary

Sensory neurons in the skin, lung, and gut directly sense immune signals and actively shape local inflammation, pain, itch, and host defense. Immune cells also alter neuronal excitability through cytokines and alarmins, forming bidirectional circuits at barrier tissues. This review organizes recent evidence into a circuit-based framework across organs and discusses how targeting neuronal components may relieve disease symptoms while affecting protective immune responses.

## 1. Introduction

Barrier tissues such as the skin, lung, and gastrointestinal tract are constantly exposed to environmental challenges while performing vital physiological functions. Classical immunological models described immune cells as the main sentinels at these interfaces, with neurons assigned to secondary sensory and reflex roles [1,2,3]. This separation reinforced the long-held belief that barrier immunity and sensory perception are parallel yet largely distinct systems.

This paradigm has now decisively shifted. Since 2020, an increasing amount of research has shown that sensory neurons, especially nociceptors and pruriceptors, serve as key regulators of barrier immunity rather than just passive responders [4,5]. Sensory neurons that innervate barrier tissues contain receptors traditionally linked with immune cells, such as Toll-like receptors, cytokine receptors, and alarmin sensors, enabling them to directly detect microbial products, allergens, and tissue injury [6].

Neuronal activation triggers the rapid release of neuropeptides like calcitonin gene-related peptide (CGRP), substance P, vasoactive intestinal peptide (VIP), and PACAP (pituitary adenylate cyclase-activating polypeptide), which have various effects on immune cells, including dendritic cells, macrophages, mast cells, innate lymphoid cells, and T lymphocytes [7]. These neuronal signals operate much faster than traditional immune cascades, allowing immediate regulation of inflammation and host defense [8,9].

Equally important is the reciprocal regulation by immune cells of neuronal function. Cytokines such as IL-33 (Interleukin-33), IL-31(Interleukin-31), IL-4(Interleukin-4), IL-13(Interleukin-13), and TNF-α (Tumor Necrosis Factor-alpha) directly modify neuronal excitability, sensitization, and desensitization, affecting pain, itch, cough, and other protective reflexes [10,11,12]. Recent studies have demonstrated that these interactions are not just modulatory but also establish stable, tissue-resident neuroimmune circuits.

Technological advances have played a key role in these discoveries. Optogenetic and chemogenetic methods have provided causal evidence that selectively activating or silencing sensory neurons is sufficient to initiate or halt immune responses in vivo [4,13]. Single-cell and spatial transcriptomic analysis has resolved spatially organized neuroimmune transcriptional programs in barrier tissues, revealing structured neuronal responses to immune challenge [14].

Notably, landmark work has shown that TRPV1^+^ (Transient Receptor Potential Vanilloid 1) sensory neurons directly regulate gut Treg abundance and phenotype, linking neuronal activity to intestinal immune homeostasis [13]. These findings emphasize that sensory neuroimmune circuits are not uniform but exhibit significant organ-specific specialization driven by local environmental cues and microbial context [1,15].

Unlike prior narrative overviews that organize findings by a mediator or organ system, this review frames sensory–immune communication as functional circuits operating at barrier tissues, progressing from stimulus detection to neuronal processing and ultimately to immune output. To strengthen mechanistic interpretation, the evidence is explicitly stratified by methodological strength. Studies employing causal perturbation approaches—such as optogenetics, chemogenetics, genetic ablation, and spatially resolved single-cell analyses published between 2021 and 2025—are prioritized when assigning immune regulatory functions to defined neuronal subtypes. In contrast, findings derived from correlative or associative approaches (e.g., transcriptomic profiling, immunostaining, or observational clinical studies) are presented as supportive but not sufficient to infer causality. The translational section remains anchored to signaling nodes that already have clinical relevance or are under active therapeutic development, with safety liabilities considered as primary constraints in target selection. Representative examples include Purinergic Receptor P2X3(P2X3) receptor antagonists for refractory or unexplained chronic cough, blockade of the CGRP signaling pathway, IL-31–directed therapies for chronic pruritus, and Nav1.8 (Voltage-gated sodium channel 1.8) channel inhibitors for acute pain management [1,13]. Barrier tissue biology has often been parsed into discrete mediators and receptors, which now looks incomplete. Accumulating data support sensory neuroimmune circuits as functional units, in which immune-derived cytokines and alarmins engage sensory neurons that, in turn, release neuropeptides capable of reshaping immune cell behavior, providing feedback. Inflammation may intensify or subside, depending on context and timing [16,17]. In this manner, neuroimmune communication is not a linear relay. Circuit-based framing allows comparisons across skin, lung, and gut without forcing false equivalence between mediators. It also exposes shared organizational logic linking itch, pain, and host defense, even when cellular participants differ. The unit of analysis shifts from molecule to circuit (Table 1).

## 2. Sensory Neurons as Immune Sentinels and Their Molecular Equipment

Barrier-associated sensory neurons function as frontline sentinels that translate environmental stimuli into coordinated immune responses [18]. In this framework, transient receptor potential (TRP) channels operate as key sensory gateways that initiate neuro-immune circuits linking external or tissue-derived stimuli to immune modulation. Among these, TRPV1 and Transient Receptor Potential Ankyrin 1 (TRPA1) represent prototypical polymodal, non-selective cation channels with high Ca^2+^ permeability that are prominently expressed on peripheral nociceptor endings and, in certain contexts, on epithelial and immune cells [19,20] (Table 2).

Structurally, these channels assemble as tetrameric transmembrane complexes with six membrane-spanning domains per subunit and cytoplasmic N- and C-terminal regulatory regions capable of integrating thermal, chemical, and inflammatory signals [21,22]. Functionally, TRPV1 detects noxious heat, acidic environments, and vanilloid ligands, whereas TRPA1 responds to reactive electrophiles [23], oxidative stress, and environmental irritants. Activation of these channels generates Ca^2+^-dependent neuronal excitation that triggers the release of neuropeptides such as calcitonin gene-related peptide (CGRP) and substance P, thereby establishing communication between sensory neurons and immune or stromal cells [24,25].

Through these pathways, TRP-expressing neurons regulate immune cell recruitment, polarization, barrier integrity, inflammatory amplification, fibrotic remodeling, pain hypersensitivity, and pruritus. The receptors discussed in this section were selected based on three criteria: strong experimental evidence linking defined neuronal subsets to immune outcomes, well-characterized expression at barrier tissues such as skin and gastrointestinal mucosa, and emerging translational relevance in inflammatory or sensory disorders (Table 2).

### 2.1. TRPV1 as a Multimodal Neuroimmune Hub

TRPV1^+^ nociceptors form a sensory–immune circuit that detects noxious thermal, chemical, and inflammatory stimuli and subsequently modulates immune activity through neuropeptide-dependent signaling pathways [18]. In this circuit, environmental or tissue-derived inputs—including heat (>42 °C), acidic microenvironments, inflammatory mediators, and exogenous ligands such as capsaicin—activate TRPV1 channels on peripheral sensory neurons [21,22]. Channel opening allows Ca^2+^ influx, which triggers neuronal depolarization and the release of effector mediators, most prominently CGRP and substance P. These mediators act on nearby immune cells, endothelial cells, and stromal elements to initiate neurogenic inflammation and shape downstream immune responses [19,25]. However, the redundancy of nociceptive signaling pathways and the widespread distribution of TRPV1 raise concerns that compensatory mechanisms and off-target effects may blunt therapeutic efficacy or produce unintended consequences during sustained pharmacological intervention (Table 2).

The functional outcome of TRPV1 activation depends on the inflammatory context. Acute activation may lead to activity-dependent desensitization through Ca^2+^/calmodulin-mediated channel closure or receptor internalization, mechanisms that underlie the clinical use of capsaicin for treating chronic pain and pruritus [25,26,27]. In contrast, persistent inflammatory mediators can lower the thermal activation threshold of TRPV1 and increase trafficking of intracellular channel pools to the neuronal membrane, thereby enhancing neuronal excitability and sustaining inflammatory signaling loops [28,29] (Table 2).

TRPV1^+^ sensory neurons are distributed across multiple organs, including the skin, gastrointestinal tract, lungs, and pancreas, where they interact with keratinocytes, immune cells, fibroblasts, adipocytes, and epithelial cells [26,30]. Through CGRP- and substance P-mediated signaling, these neurons establish tissue-specific neuroimmune circuits that regulate inflammatory responses, barrier homeostasis, and sensory perception [31,32] (Table 2).

### 2.2. Pain

In nociceptive circuits, noxious thermal or inflammatory inputs activate TRPV1^+^ dorsal root ganglion (DRG) neurons, initiating Ca^2+^-dependent signaling that links sensory detection to immune modulation. Activated neurons release CGRP, which acts on nearby immune populations to regulate inflammatory responses [19]. Within the colon, TRPV1^+^ nociceptors influence myeloid cells, macrophages, and RORγ^+^ regulatory T cells through CGRP-dependent signaling pathways, illustrating how sensory neurons participate in maintaining immune balance [20].

Persistent activation of this circuit contributes to neuroinflammation and neuronal dysfunction within the spinal cord. Mitochondrial alterations, including permeability transition pore activation and metabolic disruption, are thought to play an important role in the development of mechanical and thermal hypersensitivity. Experimental modulation of TRPV1 signaling after nerve injury has been shown to preserve mitochondrial integrity and reduce hypersensitivity [33]. Clinically, pharmacological targeting of this pathway, for example with the ultrapotent TRPV1 agonist resiniferatoxin, can produce long-lasting analgesia through functional silencing of nociceptive neurons [34]. Similar mechanisms appear to contribute to the severe pain observed in chronic pancreatitis, where inflammation-induced sensitization of TRPV1^+^ DRG neurons amplifies nociceptive signaling [35]. Despite promising preclinical and early clinical results, prolonged or irreversible TRPV1 modulation may impair protective nociception and thermoregulation, induce sensory deficits, and pose substantial safety and delivery challenges, thereby limiting its therapeutic window in humans (Table 2).

### 2.3. Fibrosis

In fibrotic disorders, TRPV1-dependent sensory circuits connect tissue injury to immune and stromal activation. Experimental models of pulmonary fibrosis demonstrate that stimulation of TRPV1^+^ neurons elevates transforming growth factor-β1 (TGF-β1) levels and alters intracellular Ca^2+^ dynamics through coordinated extracellular influx and endoplasmic reticulum release, thereby promoting fibroblast activation and migration [36].

At the molecular level, TRPV1-mediated Ca^2+^ signaling activates downstream pathways including CaMKII, calcineurin, and ERK/p38 MAPK cascades [37]. These pathways converge on TGF-β–Smad2/3 signaling in fibroblasts, facilitating myofibroblast differentiation and extracellular matrix deposition [38]. In parallel, sensory neuron-derived neuropeptides influence macrophage polarization and NF-κB-associated inflammatory responses, further shaping the fibrotic microenvironment [37,39,40] (Table 2).

### 2.4. Gut

Within the gastrointestinal tract, TRPV1^+^ nociceptors integrate luminal and inflammatory stimuli to regulate mucosal immune homeostasis. Activation of this neuronal circuit modulates colonic myeloid cells and regulatory T-cell populations through CGRP-dependent pathways that require receptor activity-modifying protein 1 (RAMP1) signaling [41]. Through these interactions, sensory neurons contribute to the maintenance of epithelial barrier integrity and immune balance.

In both inflammatory and functional gastrointestinal disorders, TRPV1-positive sensory fibers are significantly increased, with some studies reporting up to a 3.5-fold elevation compared with healthy tissue. Importantly, this expansion can occur even in the absence of overt inflammation, suggesting that TRPV1-mediated neuronal circuits may contribute to visceral hypersensitivity and altered immune signaling in these conditions [42] (Table 2).

### 2.5. Itch

Pruriceptive circuits also rely on TRPV1-dependent signaling to integrate itch-inducing stimuli. Many itch-responsive sensory neurons co-express histamine H1 receptors and TRPV1 channels. Activation of histamine receptors initiates intracellular signaling cascades that converge on TRPV1 activation, leading to membrane depolarization and Ca^2+^-dependent neuronal excitation that drives itch perception [43,44,45,46,47] (Table 2).

In addition to histaminergic pathways, TRPV1 participates in non-histaminergic itch circuits involving receptors such as protease-activated receptor 2 (PAR2), Toll-like receptor 7, and vesicular glutamate transporters. Through partial overlap with TRPA1 signaling pathways, these circuits coordinate neuronal and immune responses that sustain chronic pruritus [48,49]. Consistent with this role, genetic deletion of TRPV1 reduces inflammatory hyperalgesia and itch behaviors, while clinical studies demonstrate that topical capsaicin can alleviate pain and pruritus by inducing functional desensitization of TRPV1^+^ sensory neurons [50] (Table 2).

### 2.6. Nav1.8 and Pain

Nav1.8 is mainly expressed in primary sensory neurons and influences electrogenesis by driving repetitive action potential (AP) firing. Nav1.8 (along with Nav1.9) demonstrates strong pharmacological resistance to low levels of tetrodotoxin (TTX) and exhibits depolarized voltage dependence of inactivation [51]. Some studies demonstrated the role of the alteration of Nav1.8 expression in chronic pain. For example, the expression of Nav1.8 in the masseter muscle-innervating nerves is increased in patients with chronic myofascial temporomandibular pain [52]. Evidence linking SCN10A (Sodium Voltage-Gated Channel Alpha Subunit 10) variants to nociceptive signaling has largely emerged from genetic and functional association studies. Early observational analyses suggested that alterations in SCN10A may influence neuronal excitability and pain susceptibility. In a notable genetic study, Faber et al. identified three SCN10A polymorphisms associated with peripheral neuropathy. Functional characterization demonstrated that two variants, L554P and A1304T, significantly enhanced the responsiveness of Nav1.8 channels to membrane depolarization, thereby increasing the excitability of the corresponding sensory neurons. Although these findings are primarily associative, they provide strong mechanistic support for the hypothesis that SCN10A variants contribute to abnormal neuronal excitability and pain phenotypes [53]. Furthermore, evidence shows that Nav1.8 may influence other key factors that impact nociception and pain perception. For example, in a mouse model of psoriasis, Nav1.8-bearing sensory neurons were found to interact with antigen-presenting cells to influence the expression of specified cytokines (interleukin-12/interleukin-23) [54]. While Nav1.8 expression has not been demonstrated in a healthy human brain, these channels have been identified in Purkinje fibers from the cerebella of rodents used in multiple sclerosis (MS) models, as well as those extracted from post-mortem patients diagnosed with MS [55]. Recent studies presented an important role of Nav1.7–1.9 in itch signaling. Case studies explained that gain-of-function mutations in these Nav channels can cause paroxysmal itch in affected patients [56]. Studies have reported optimistic outcomes for prescribing Suzetrigene in controlling moderate to severe pain via inhibition of the Nav1.8 signals [57,58]. The process of developing Nav1.7 and Nav1.8 inhibitors is hampered by drug intolerance, insufficient target occupancy, and off-target, unwanted side effects. Many research investigations, including those from both animal- and human-based studies, support the fact that Nav1.8 is a target for controlling pain. New concentration studies are essential to investigate the exact underlying mechanism and impact that this channel has in relation to pain. Additionally, further research needs to be carried out to develop pharmacological agents and techniques that are more selectively targeted for Nav1.8. Currently, as we improve our ability to manipulate this channel and harness its functionality, we will have an opportunity to provide patients with novel, more potent and safer analgesic medications (Table 2).

### 2.7. MrgprA3 (Mas-Related G Protein–Coupled Receptor) and PAIN

It has been reported that MrgprA3 is expressed highly in the dorsal root ganglia [59]. Using single-cell RNA (Ribonucleic Acid) sequencing of DRGs, DS George etal demonstrated an increased expression of the Mrgpr in a subpopulation of DRG neurons in the HFD mouse and human model of painful diabetic neuropathy [60] (Table 2).

### 2.8. MrgprA3 and ITCH

Several subtypes of MRGPRs (Mas-Related G Protein-Coupled Receptors) are expressed exclusively in primary sensory neurons, indicating that they play an important role in sensory processes, including itch [61]. MrgprA3, a subtype of MRGPR, mediates itch induced by the antimalarial drug chloroquine. Ablation of MrgprA3^+^ neurons reduces chloroquine-evoked itch, as well as responses to other pruritogens. Notably, mice expressing TRPV1 exclusively in MrgprA3^+^ neurons display itch rather than pain when treated with capsaicin [62]. These findings suggested that MrgprA3^+^ neurons play a specific role in the transmission of itch signals. Activation of MrgprA3 and MrgprA3^+^ neurons has been demonstrated to contribute to itch associated with dry skin and contact dermatitis [63]. More recent work has begun to clarify neuroimmune mechanisms underlying itch associated with immune checkpoint inhibitors. Lixuan Li et al. reported that repeated administration of Nivolumab upregulates IL-2Rβ/γ expression in MrgprA3^+^ sensory neurons through activation of the SHP-1–JNK–STAT5 signaling pathway. This neuronal sensitization occurs in parallel with elevated systemic IL-2 levels, together forming a neuroimmune feedback loop that amplifies pruritic signaling. These findings provide mechanistic insight into how immune checkpoint blockade can indirectly modulate sensory neuron activity and contribute to checkpoint inhibitor–induced pruritus [64]. Future studies should concentrate on targeted blockade of MrgprA3 in controlling pain and itch. MrgprA33 regulates IL-17^+^ γδ T cell expansion and shaping cytokine expression in cutaneous antigen-presenting cells (APC). MrgprA3 neuron activation downregulates interleukin 33 (IL-33) but induces IL-1β and TNFα in macrophages and cDC2 partially through the neuropeptide CGRP. Macrophages exposed to MrgprA3-derived secretions or bearing cell-intrinsic IL-33 deletion show increased chromatin accessibility at multiple inflammatory cytokine loci, promoting IL-17/23-dependent changes to the epidermis and anti-helminth resistance [4] (Table 2).

## 3. Key Molecular Mediators and Intercomes

Sensory neuropeptides constitute a major efferent arm of neuroimmune communication, translating nociceptor and pruriceptor activation into coordinated inflammatory, stromal, and neuronal responses. Among these mediators, calcitonin gene–related peptide (CGRP), substance P, vasoactive intestinal peptide (VIP), and pituitary adenylate cyclase–activating polypeptide (PACAP) represent well-characterized signaling molecules released from peripheral and central terminals of primary afferents. These peptides act through distinct G protein–coupled receptors yet converge on Ca^2+^-dependent and transcriptional pathways that shape pain processing, itch generation, immune cell recruitment, and tissue remodeling. This section provides a structured overview of their molecular biology, signaling mechanisms, and context-dependent functions, establishing the framework for the downstream subsections that detail their roles in pain, itch, and neuroimmune regulation.

### 3.1. CGRP and Pain

CGRP, a 37-amino-acid neuropeptide exhibiting diverse biological roles in both peripheral tissues and the central nervous system [65]. To what extent CGRP is involved in non-headache pain conditions is not fully clarified and whether CGRP antagonism may represent a useful therapeutic approach for the treatment of chronic pain is unknown. The highest concentrations of CGRP have been found in the outer laminae of the spinal cord dorsal horn and in the trigeminal nucleus caudalis (TNC), which correspond to the central terminals of primary afferent neurons with soma in the DRG and TG, respectively [66]. In contrast to purely associative observations, causal circuit-level evidence has been provided by functional neurophysiological studies. Kang et al. demonstrated that calcitonin gene-related peptide (CGRP)–expressing neurons in the parvicellular subdivision of the subparafascicular nucleus (SPFp) play a direct role in pain processing. Anatomical tracing revealed monosynaptic input from the spinal dorsal horn to SPFp CGRP neurons. Importantly, in vivo calcium imaging showed robust activation of these neurons in response to mechanical, thermal, and inflammatory stimuli, providing functional evidence that this neuronal population participates directly in nociceptive circuit signaling [67]. These CGRP receptors may promote the release of glutamate, acting in opposition to the inhibitory effect of the α_2_-adrenergic receptors, but the fact that some of these neurons also express the enkephalins is a confounding factor [68]. Activation of the CGRP receptor with the release of the neuropeptide from primary afferent terminals leads to activation of signaling pathways that also sensitize the NMDA receptor [69]. RONS/TRPA1/CGRP axis is known to be the most crucial part in the pathogenesis associated with migraine and pain syndrome. Epigenetic alterations in the genes encoding the components of the antioxidant system RONS/TRPA1/CGRP axis is the major contributing factor for the pathogenesis, prevention, and treatment associated with migraine. On the contrary, being a sequence-specific axis, only non-coding RNA could be the potential target for the treatment associated with migraine based on the epigenetic profile for the RONS-TRPA1-CGRP axis [70].

### 3.2. CGRP and ITCH

It also regulates the function of immune cells in general, also in dendritic cells [71]. There are also many studies which show the increased numbers of CGRP-immunoreactive nerves in lesional skin in AD patients compared to the non-lesional skin, and mast cell–nerve fiber contacts [72]. GPCR recognizes many kinds of itching chemical stimuli. Multiple G protein–coupled receptors (GPCRs), including histamine receptors (H1R, H4R), protease-activated receptor 2 (PAR2), Mas-related G protein–coupled receptors (MRGPRs), cysteinyl leukotriene receptors (CysLTRs), and endothelin receptors (ETA and ETB), have been implicated in neuroimmune signaling. The G-protein-coupled signaling pathway is activated through phospholipase C or phospholipase Gβγ. Therefore, it transduces the activation of the TRP cation channels [18,73]. In the W Ding experiment, when the pDMECs were exposed to the CGRP and then co-cultured with the LCs, CD^4+^ T cells, and the Ag, it increased the levels of IL-6 and IL-17A, simultaneously suppressing the production of IFN-γ, IL-4, and IL-22 compared to those in the pDMECs exposed to medium only [74]. These inflammatory mediators are increased in the chronic dermatologic disease of psoriasis and chronic itching.

### 3.3. Substance P and Pain

**Figure 1:**
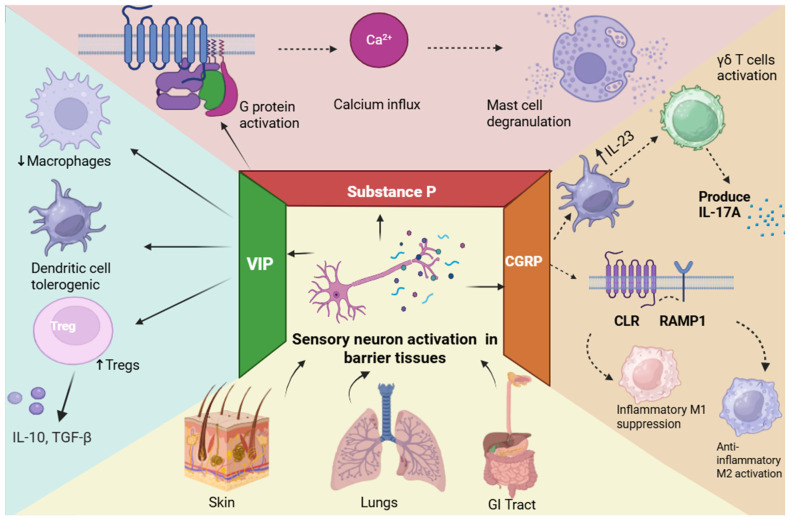
**Neuropeptide-mediated sensory neuron–immune crosstalk at barrier tissues.** Sensory neurons at barrier sites (skin, lung, and gastrointestinal tract) regulate immune responses through the release of neuropeptides. Upon activation, neurons secrete substance P, calcitonin gene-related peptide (CGRP), and vasoactive intestinal peptide (VIP), each engaging distinct receptor pathways and immune programs. Substance P activates G protein–coupled receptors to induce Ca^2+^ signaling and mast cell degranulation. In contrast, CGRP, acting through the CLR–RAMP1 complex, suppresses pro-inflammatory macrophage activity and promotes M2-like polarization, while also shaping inflammatory cascades that can indirectly drive γδ T cell activation and IL-17A production via cytokines such as IL-23. VIP exerts immunoregulatory effects by limiting macrophage activation, inducing tolerogenic dendritic cells, and enhancing regulatory T cell responses. Together, these pathways coordinate neuroimmune interactions that balance inflammation and tissue homeostasis. Created by the authors using www.BioRender.com (accessed on 21 December 2023). No copyrighted material was reproduced.

Substance P is particularly concentrated in the dorsal horn of the spinal cord, the substantia nigra, and the amygdala [75]. Among the neurokinins, Substance P demonstrates exceptional avid potency upon binding to NK1 receptors, whereas neurokinin A and neurokinin B show much more deliberation toward NK2 and NK2, respectively. When substance P binds to the Gq-coupled NK1 receptor, the G-protein becomes activated (Figure 1), which further activates phospholipase-CB. Phospholipase-CB converts PIP2 into IP3 and DAG. In turn, IP3 acts on the endoplasmic reticulum, causing calcium to be released into the cytosol [76]. Additionally, the activation of this cascade also leads to the up-regulation of cytokines, transcription factors, and nuclear factor kappa-light-chain-enhancer of activated B cells (NF-κB), which stimulates pain [77]. It does so through Substance P contained in the dendritic shaft and spine vesicles projecting to form axodendritic connections. The spread of the stimuli to the dendritic spines triggers the release of Substance P. In turn, Substance P can act locally or through volume transmission on the neurons in the body [78]. Furthermore, Substance P induces an increase in NMDA-activated depolarization of neurons, vital for developing algesia [79].

### 3.4. Substance P and ITCH

However, there are some shortcomings in these theories related to the impact of Substance P on sensitization, the augmentation of glutamate, and the expansion of receptive fields. One such major shortcoming is that while the impact of Substance P has been known and identified, the mechanism for this impact is not known [80]. Its function within keratinocytes, fibroblasts, and mast cells seems to be mainly cholinergic and involves triggering inflammation and has been described as involving short-term vasodilation and mast cell degranulation; it also involves nerve growth factor production within keratinocytes and neurogenic inflammation related to erythema, wheals, and pruritus (Figure 1) [81]. SP has been shown to induce skin inflammation by promoting the expression of nerve growth factor and leukotriene B4 in keratinocytes and the secretion of pro-inflammatory mediators from mast cells [82]. SP-stimulated MRGPRX2-KI PMCs also released high levels of histamine and mast cell protease 4 (MCPT4) chymase [83]. To develop antipruritic therapies, it is important to understand similarities and differences across pruritic diseases.

### 3.5. VIP and PACAP

Both the central nervous system and peripheral tissues, including barrier regions where neuroimmune interactions take place, express the neuropeptides VIP and PACAP. Potential therapeutic targets, these peptides communicate via G protein-coupled receptors (PAC1, VPAC1, and VPAC2). They have been shown to have both pro- and anti-nociceptive effects on pain regulation in the brain and spinal cord, and the underlying signaling mechanisms are still unclear [84]. On the other hand, VIP primarily performs immunoregulatory tasks in peripheral tissues, such as promoting regulatory T cell responses, inducing tolerogenic dendritic cells, and suppressing pro-inflammatory macrophage activation, as illustrated in Figure 1. These context-dependent effects support the inclusion of VIP and PACAP in neuropeptide-mediated immune regulatory pathways and emphasize their dual roles throughout neuroimmune compartments [85].

### 3.6. GALANIN and PAIN

Galanin is a neuroendocrine neuropeptide involved in pain and reinforces the analgesic action of morphine [86,87]. Further, it is proposed that endogenous galanin regulates normal conditions, this peptide is found primarily in the dorsal root ganglia. the DRG and superficial layers of the spinal dorsal horn, respectively [20]. Currently, four peptides are considered to belong to the galanin family: galanin-message, galanin-like peptide, alarin and spexin [88]. Galanin acts via three receptor subtypes: GalR1-3. Under normal conditions, galanin is thought to play a minor role in nociception; however, it increases after injury, particularly in the DRG and spinal cord, in which it plays a mostly antinociceptive role [89]. There were complex aspects of the involvement of the galanin system in pain. Initial research showed the inhibitory and, at a certain dosage, excitatory effects of galanin, together with increased inhibition following nerve damage [90]. The signal pathways of the three GALR subtypes are quite different, and the signal transduction mechanism for analgesic effects for the inflammatory pain of galanin/GALR has not been fully explained. CaMKII and PKC represent a family of serine/threonine protein kinases that are all PLC-dependent, Ca^2+^-related protein kinases. Each of the many PKC isoforms plays an important role in the development of central and/or peripheral sensitization in various persistent pain conditions [91]. Earlier neurochemical studies also suggested a modulatory role for neuropeptides in trigeminal pain pathways. Wang et al. investigated the function of the neuropeptide galanin in orofacial nociception and reported that galanin signaling modulates nociceptive transmission within trigeminal circuits. Although these findings are primarily associative, they indicate that alterations in galanin expression or receptor activity may influence the initiation and persistence of orofacial pain states, highlighting galanin as a potential regulatory component of trigeminal pain processing [92]. By contrast, there are several galanin ligands that display a non-selective effect towards GalR1/R2 receptors and exert antinociception [93]. In addition, future research might be aimed at exploring signaling transduction pathways of galanin receptors (GalR1-3) under inflammatory and neuropathic pain conditions, especially on Ca^2+^ cascade related to PLC-dependent Ca^2+^ kinases.

### 3.7. GALANIN and ITCH

Galanin takes part in the itching mechanism. Spinal inhibitory interneurons, including galanin+ cells, interacting with Bhlhb5+ and dynorphin+ cells, are also part of the inhibitory pathway as they directly inhibit GRPR+ cells [94]. Spinal inhibitory interneurons, including galanin+ and nNOS+ cells, mainly connect with GRPR+ cells, mediating the chemical itch [95]. Hence, future studies should focus on Galanin as a therapeutic issue in controlling chronic itch.

## 4. Mechanisms of Bidirectional Neuroimmune Signaling

**Figure 2:**
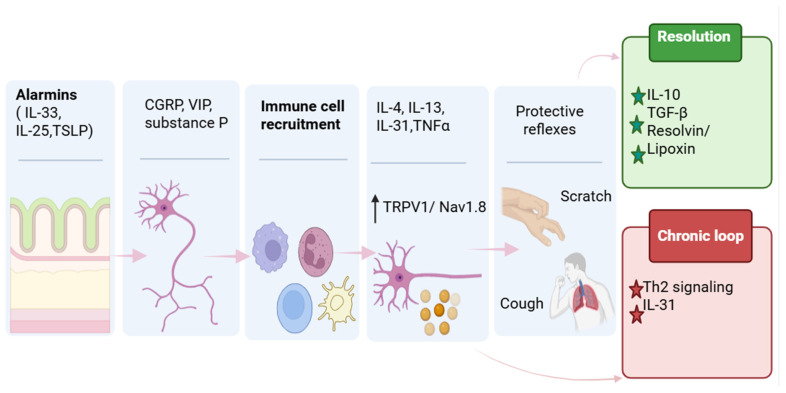
**Stepwise activation of the sensory neuroimmune circuit and divergence toward resolution or chronic inflammation.** Barrier-derived alarmins (IL-33, IL-25, and TSLP) initiate activation of sensory neurons, leading to the release of neuropeptides including CGRP, VIP, and substance P. These mediators drive immune cell recruitment and activation, establishing bidirectional signaling between neurons and immune cells. Subsequent cytokine feedback (IL-4, IL-13, IL-31, and TNF-α) further sensitizes neuronal pathways through TRPV1 and Nav1.8, amplifying neuroimmune responses. This signaling cascade elicits protective behavioral reflexes such as scratching and coughing. The circuit then diverges toward either resolution, mediated by anti-inflammatory and pro-resolving signals (IL-10, TGF-β, resolvins, and lipoxins), or persistence of a chronic inflammatory loop driven by Th2 signaling and IL-31. This framework highlights the dynamic balance between protective host responses and maladaptive neuroimmune amplification. Created by the authors using www.BioRender.com (accessed on 21 December 2023). No copyrighted material was reproduced.

The nervous and immune systems both monitor internal and external conditions and coordinate responses that preserve physiological homeostasis [96]. Neuroimmunology is based on the concept that these two systems communicate bidirectionally. From an immune function perspective, neuro–immune interactions can influence immunity through multiple modes of regulation, producing diverse functional outcomes. Figure 2 summarizes bidirectional signaling connections with barrier-resident immune cells across skin and lung.

### 4.1. Neuron-to-Immune Signaling

Neuron-to-immune signaling starts with activity in peripheral afferents and ends with local effector delivery into immune and stromal microenvironments. At barrier surfaces, TRPV1 and TRPA1 provide major entry points for noxious heat and reactive chemical ligands [16,97], while TRPV4 contributes to mechanochemical and osmotic sensing in airway and other tissues [98], and TRPM8 links cold and menthol-responsive signaling to distinct sensory programs [99]. Mechanosensitive channels such as Piezo2 and pruritogen-sensing MRGPRs add mechanical and GPCR-driven channels into the same excitability module. Once receptor potentials reach threshold, Nav1.7 shapes firing probability and recruitment, and Nav1.8 supports sustained firing under inflammatory conditions. The following examples illustrate how these transduction modules couple neuronal activity to mediator release and immune modulation.

TRPV1 contributes to neuron-to-immune communication through mechanisms that couple channel activation to intracellular signaling and mediator release. TRPV1 activation drives extracellular Ca^2+^ influx and engages Ca^2+^-responsive signaling pathways. Beyond sensory neurons, TRPV1-mediated cation influx has been demonstrated in dendritic cells, T cells, macrophages, and microglia, where it can regulate apoptosis, migration, cytokine production, inflammasome-linked programs, and inflammatory polarization [100,101,102,103]. Downstream signaling can involve CaMKII–Nrf2 and CaMKK2/AMPK/mTOR pathways, whereas pharmacologic or genetic TRPV1 inhibition perturbs Ca^2+^ homeostasis and can induce mitochondrial stress [39,104]. In nociceptors, TRPV1 activation also links electrical activity to neuropeptide release, providing an additional route through which TRPV1 shapes immune responses at barrier tissues. In sensory neurons, TRPV1^+^ nociceptors can regulate immunity through activity-dependent neuropeptide signaling. An unbiased chemogenetic screen provides causal evidence that sensory neuron activity can directly instruct barrier immunity in vivo. Activation of defined gut-innervating neuronal classes revealed that TRPV1^+^ nociceptors exert the broadest immune effects, including reduced colonic myeloid populations and down-regulation of RORγ^+^ regulatory T (Treg) cells, with associated Treg transcriptional changes and decreased proliferation. Neuroanatomical gain- and loss-of-function approaches localized this phenotype to TRPV1^+^ DRG neurons, not vagal TRPV1^+^ neurons, underscoring pathway-specific immune control. Mechanistically, the effect required CGRP and its receptor component RAMP1, defining a ligand–receptor axis through which nociceptor activity can gate Treg programs at barrier surfaces [13].

In addition to CGRP, other nociceptor-derived neuropeptides can shape immune responses through receptor-specific signaling. As a neuropeptide released from activated nociceptors, substance P shows how receptor choice can gate immune consequences. Canonically, substance P signals through NK1R, and modulation of the substance P–NK1R axis is associated with pro-inflammatory cytokine programs, including IL-1β and TNF-α. In myeloid-lineage immune cells, including macrophages, NK1R-linked signaling can engage Ca^2+^-dependent pathways and converge on NF-κB–associated inflammatory outputs, consistent with a mechanism by which neuron-derived substance P shapes barrier inflammation [105,106,107,108]. In T cells, NK1R-linked Ca^2+^ flux can engage NFAT and NF-κB pathways that support survival and effector functions [109]. VIP provides a neuron-derived signaling axis whose immune consequences depend on the local distribution of VIP receptors across immune and epithelial compartments. In the intestine, neuronal VIP can act directly on VIPR2-expressing innate lymphoid cells, including ILC2 and ILC3, and increase type 2 and type 3 effector outputs after feeding, consistent with a neuron-to-immune pathway that modulates barrier immunity through defined receptor–ligand logic [110]. Additionally, neuroepithelial VIP–VIPR1 signaling can regulate epithelial programs that set the downstream immune context, such that the same VIP pathway can shift host defense outputs in a challenge-dependent manner [111]. These effects often depend on epithelial relay mechanisms that translate neuronal signals into local cytokine and barrier programs.

Many barrier circuits operate through epithelial intermediaries, which function as active relays rather than passive targets. In the intestine, acetylcholine released from enteric neurons regulates goblet cell-associated antigen passages (GAPs) via region-specific muscarinic receptors, and higher-intensity cholinergic signaling can additionally trigger mucus secretion [112,113]. Nociceptor-derived CGRP can signal directly to goblet cells expressing CALCRL/RAMP1 to increase mucus layer thickness and support barrier protection, and can limit GP2^+^ M cell density in follicle-associated epithelium, thereby constraining pathogen translocation [114].

Across barrier tissues, shared sensory-neuron activation modules can be coupled to distinct effector pathways that reflect local stromal and immune organization. In skin, nonpeptidergic MRGPRD-expressing sensory afferents tonically suppress mast-cell reactivity via glutamate signaling, supporting a neuron-to-immune mechanism that constrains cutaneous inflammation [115]. In acute tissue injury, Nav1.8-lineage nociceptors signal through CGRP to shape neutrophil and macrophage responses that support tissue repair, indicating that nociceptor-derived neuropeptides can also instruct pro-repair immune programs in the skin [116]. In the gut, TRPV1-lineage nociceptors direct goblet-cell mucus production through CGRP signaling via epithelial RAMP1, strengthening barrier protection in colitis-relevant settings [117]. In parallel, disruption of gut nociceptor signaling reduces substance P availability, and exogenous substance P can restore tissue-protective outputs in a microbiota-dependent manner, supporting a second neuropeptide route for neuron-to-immune control of barrier defense [118]. In the lung, pulmonary neuroendocrine cells act as an epithelial relay with CGRP and GABA signaling capacity and a broad neuropeptide repertoire, and eosinophil extracellular traps can activate this neuroendocrine axis in allergic airway inflammation [119].

Sustained peripheral afferent activity can propagate neuroimmune signaling into central relay sites, where immune-like glial programs influence nociceptive processing. In peripheral nerve injury models, ongoing afferent drive into the spinal dorsal horn is associated with microglial activation and a shift toward reactive pro-inflammatory microglial states. Mechanistically, this is supported by stress- and kinase-signaling cascades such as ASK1–JNK/p38 MAPK, which promote neuroinflammatory activation, together with TRAF6-dependent c-JUN and NF-κB signaling that cytokine-inductive microglial states. As a result, microglia increase production of pro-inflammatory cytokines including IL-1β, IL-6, and TNF-α, providing a plausible route by which persistent nociceptor input can amplify central sensitization [120,121]. Thus, nociceptor activity initiated at barrier tissues can extend beyond local microenvironments to recruit immune-like effector programs within central relay circuits. These tissue-resolved examples are expanded in Section 5, where skin, lung, and gut circuits are discussed in parallel to highlight shared neuron-to-immune modules and tissue-specific effector pathways [122] (Table 3).

### 4.2. Immune-to-Neuron Signaling

Reciprocal signaling from immune and stromal cells back to sensory neurons regulates excitability and shapes pain and itch outputs in inflamed barrier tissues. Immune-to-neuron signaling operates through two major modes: direct activation and sensitization. Direct activation produces immediate Ca^2+^ influx and firing, while sensitization lowers thresholds and amplifies responses to other stimuli, commonly by converging on shared kinase nodes and TRP channel coupling [122].

IL-4 and IL-13 can signal through IL-4Rα (type I or type II receptor complexes) on sensory neurons, recruiting JAK-family kinases and activating STAT6-linked transcription; PI3K–AKT signaling and IRS-associated routes run in parallel. These pathways can shift excitability quickly through post-translational effects on channel gating and trafficking, and more slowly through transcription that stabilizes membrane availability for voltage-gated sodium channels and TRP channels [126,127]. Cytokine-driven persistence in sensory phenotypes also has precedent at the level of translation control: IL-31-receptor-linked itch requires sensory neuronal STAT3, and MNK–eIF4E signaling has been tied to durable sensory neuron states after injury paradigms [11,128].

These modes often coexist, with outcomes shaped by mediator concentration, receptor density, and neuronal state. At barrier surfaces, immune mediators tune nociceptor and pruriceptor excitability via receptor-ion channel coupling, rapidly increasing gain through shared kinase nodes and voltage-gated channel trafficking [129]. In itch-dominant circuits, cytokines can act as primary neuronal inputs: IL-31 signaling requires intact neuronal IL-31 receptor gene programs and downstream STAT3 in sensory neurons, establishing a cell-intrinsic pathway for cytokine-evoked pruriceptor activation [11]. Beyond classical cytokines, immune circuits can also set neuronal thresholds in a priming mode: epidermal γδ T cell–derived IL-3 acts on IL-3Rα–expressing sensory neurons via JAK2 and STAT5 to lower the activation threshold to allergens without necessarily triggering itch on its own [130]. Finally, type 2 networks can reshape neuronal programs at tissue level; recent work supports an IL-4 and IL-13 to IL-4Rα axis that modulates cutaneous sensory nerve architecture and neuroimmune coupling, offering a mechanistic basis for why chronic type 2 inflammation is tightly linked to persistent itch [131]. Related cytokine-driven mechanisms also sensitize nociceptors, linking inflammatory mediator exposure to pain hypersensitivity. In peripheral pain pathways, inflammatory cytokines can increase nociceptor excitability through rapid signaling that modifies ion-channel function and membrane availability. IL-1β can act directly on DRG neurons to increase excitability through IL-1 receptor–dependent mechanisms, including reduced function of large-conductance Ca^2+^-activated K^+^ channels and consequent changes in firing properties after prolonged exposure [132]. In addition, tissue injury can induce activity-dependent cleavage of neuronal IL-1β within DRG neurons, and the cleaved form is sufficient to promote sensory neuron activation and pain hypersensitivity, supporting a neuron-intrinsic route by which IL-1β contributes to peripheral pain signaling [133]. TNF-α can also produce rapid sensitization through kinase-linked control of voltage-gated sodium channel trafficking. A recent study showed that acute TNF-α exposure regulates Nav1.7 channel insertion in a compartment-specific manner in sensory neurons, providing a direct mechanism for increased excitability that does not require changes in stimulus intensity [129]. A second component is slower remodeling, where cytokines engage transcriptional and translational programs that stabilize sensitization. In human DRG nociceptors, IL-6 has been shown to induce nascent protein synthesis primarily through MNK1/2–eIF4E signaling, providing evidence that gp130-associated cytokine signaling can drive protein synthesis programs in human sensory neurons that are relevant to persistent hyperexcitability [134]. IL-17A is well supported as a sensitizing cytokine that can couple type 17 inflammation to TRP channel–dependent nociceptor output. In a mechanistic in vivo study, macrophage-derived IL-23 promoted IL-17A signaling that acted on nociceptors and required TRPV1 to drive mechanical pain, establishing a defined immune-to-neuron pathway in which IL-17A functions upstream of TRPV1-dependent transduction [135].

Effective immune-to-neuron signaling is further shaped by changes in vascular permeability and cellular proximity that increase immune access to peripheral axons and sensory ganglia. After peripheral nerve injury or inflammatory challenge, immune access to peripheral axons and sensory ganglia increases because vascular and extracellular-matrix constraints become more permissive. In vivo, matrix remodeling is one mechanism that can raise endoneurial vascular permeability and support leukocyte entry, with MMP-9 activity highlighted as a contributor to barrier disruption and macrophage migration in peripheral nerve compartments [136]. At the ganglion level, inflammatory conditions can also increase blood–DRG barrier permeability and promote close macrophage surveillance of neuronal somata, creating a physical interface for cytokine and lipid signaling into excitability programs [137]. Comparable neuroimmune signaling is also observed at meningeal trigeminovascular interfaces, where neuropeptide and immune mediator release can be experimentally quantified [138,139]. Moreover, peripheral glia can shape the entry and positioning of myeloid cells. For example, Schwann cell programs can promote macrophage recruitment in injured nerves through defined cytokine–receptor axes [140]. Consistent with a broader glial contribution to matrix-linked signaling, satellite glial cells have also been reported to express MMP-9 in sensory ganglia in neuropathic contexts [141]. Once established at this interface, macrophages provide a major source of mediators that sustain or resolve nociceptor sensitization. In sensory ganglia, neuron-associated macrophages can proliferate after nerve injury and act as a local source of pro-inflammatory cytokines, including TNF and IL-1β, with CX3CR1-linked signaling implicated in macrophage expansion and pain development [142,143]. In addition to cytokines and chemokines, macrophage-derived trophic signals can tune baseline sensory responsiveness. Recent genetic evidence supports a pathway in which dermal, nerve-associated macrophages regulate tissue NGF availability through SNX25-dependent control of Nrf2 stability, thereby setting peripheral sensory sensitivity in vivo [144].

Macrophages can also adopt pro-resolving states that reduce neuronal gain and support repair. In a chemotherapy-induced neuropathic pain model, a CD8 T cell–IL-13 pathway increased IL-10 production by DRG macrophages, and this macrophage IL-10 axis was required for normal pain resolution [145]. Spinal macrophage programs also promote recovery from neuropathic pain after peripheral nerve injury, supporting a central component of macrophage-mediated resolution [146]. Macrophage-derived opioid peptides can reduce nociceptor drive in inflammatory settings, indicating that analgesic outputs can arise from macrophage activation programs in addition to neuronal mechanisms [147].

In parallel with cytokine-driven nociceptor sensitization and resolution, several pruritogen pathways directly activate or sensitize peripheral pruriceptors. Histaminergic itch involves direct activation of sensory neurons. Histamine signals through H1R and requires TRPV1 to drive Ca^2+^ entry and pruriceptor firing. In mice, disrupting TRPV1 reduces histamine-evoked currents and scratching, supporting TRPV1 as a key downstream effector [147]. A later study showed that post-translational regulation of TRPV1 alters histaminergic itch, supporting functional coupling between H1R signaling and TRPV1 activity [148].

Protease-driven itch is typically non-histaminergic. During epithelial stress, dysbiosis, or inflammation, proteases can activate PAR2 and increase primary afferent excitability. A recent in vivo study showed that Acute gut microbiota perturbation increased DRG neuron excitability through circulating protease activity, and this effect was reduced by PAR2 pathway disruption, supporting protease–PAR2 signaling as a rapid input to sensory neurons [149]. Intrathecal PAR2 agonist studies in rodents show that PAR2 activation modulates DRG neurons and spinal processing, supporting a role for PAR2 signaling in somatosensory excitability [150]. In tissue-engineered human skin models, PAR2-related itch signaling co-occurs with TRP channel expression, supporting convergence of protease sensing on TRP-dependent excitation in peripheral afferents [151].

TSLP functions as an epithelial-to-neuron pathway that amplifies inflammation and promotes sensory sensitization. In airway epithelium, TRPV1/TRPA1-driven Ca^2+^ influx activates calcineurin–NFAT signaling, inducing TSLP and other cytokines, linking epithelial Ca^2+^ signaling to neuroimmune output magnitude [152]. In chronic itch, IL-4 and TSLP programs correlate with increased DRG TRPA1 expression and enhanced scratching, indicating TRPA1-dependent sensitization [153]. Overall, TSLP can amplify TRP-linked neuronal gain during barrier inflammation.

Cytokine pruritogens differ in direct activation versus sensitization. IL-31 acts as a direct pruritogen because IL-31 signaling requires sensory neuron–intrinsic receptor programs, and neuronal STAT3 supports IL-31 receptor expression and itch [11]. In contrast, IL-4 and IL-13 act primarily as sensitizers; brief pre-exposure increases neuronal Ca^2+^ responses to histaminergic and non-histaminergic pruritogens in human DRG cultures and induces transcriptional changes consistent with sustained excitability remodeling [10]. Recent work also supports an IL-4/IL-13–IL-4Rα axis as a regulator of inflammatory skin innervation patterns and itch responsiveness, consistent with a mechanism in which chronic type 2 inflammation sustains itch partly by remodeling peripheral sensory architecture in addition to acute channel gating [131].

IL-33 signals through a receptor complex containing ST2 and IL-1RAcP, recruiting MyD88, IRAK1, IRAK4, and TRAF6 to activate NF-κB and MAPK pathways. In sensory neurons, IL-33 can increase excitability by suppressing A-type K^+^ currents via ST2-dependent Syk and p38 signaling, but neuronal ST2 contributions vary across contexts [154,155].

The cellular locus of IL-33 signaling in itch varies across models, affecting the interpretation of IL-33–ST2 pathways in immune-to-neuron mechanisms. In a dry-skin chronic itch model, conditional deletion of ST2 in sensory neurons reduces itch, supporting a neuron-intrinsic requirement in specific barrier settings [156]. In histaminergic itch paradigms, IL-33 enhances scratching through immune-cell ST2 signaling; mast cells are prominent IL-33R-expressing cells, and IL-33–activated mast cell programs increase histaminergic itch via IL-13, while neuronal IL-33 signaling is dispensable [157].

Immune-to-neuron signaling includes central integration, where inflammatory cues produce durable changes in dorsal horn gain via microglia–neuron coupling. Similar microglia-dependent gain control has been demonstrated in trigeminal nociceptive relays in chronic migraine models [158]. In neuropathic and inflammatory pain, extracellular neuropeptides engage microglial P2 receptors. P2Y12 signaling activates an SFK–p38 cascade that drives IL-18–dependent spinal sensitization, promoting neuronal hyperexcitability [159]; SFK-dependent purinergic signaling has also been implicated in cortical spreading depolarization–associated neuroinflammation via P2X7 in migraine models [160]. Microglial inflammatory signaling also drives sensitization in trigeminocervical complexes in chronic migraine models [161]. Additionally, increased microglial P2X4 surface expression is sufficient to promote spinal neuron hyperexcitability and tactile allodynia, indicating ATP-gated microglial control of dorsal horn output [162]. Beyond soluble mediators, microglia can stabilize persistent pain by selective removal of dorsal horn synapses after peripheral nerve injury, providing a structural route for long-lived circuit remodeling [163]. A complementary mechanism involves microglia-derived BDNF acting via TrkB to promote KCC2 ubiquitination and hypofunction in the dorsal horn, weakening inhibitory control and thereby amplifying nociceptive transmission [164]. These findings link peripheral inflammation to central sensitization and identify microglia as intermediaries shaping sensory processing in central relay sites.

Pro-resolving signals counterbalance cytokine-driven sensitization and restore sensory set-points, not only by reducing inflammation but by actively resetting excitability. Specialized pro-resolving mediators (SPMs) such as resolvins limit inflammatory amplification in peripheral nerves and spinal cord by attenuating inflammasome- and MAPK-linked pathways. In neuropathic pain models, resolvin D1 acting via ALX/FPR2 suppresses NLRP3–ERK signaling in DRG and spinal tissues and reduces mechanical allodynia [165]. RvD1 has been reported to inhibit microglial activation and BDNF–TrkB signaling, supporting a central mechanism by which resolution limits dorsal horn sensitization [166]. At primary afferents, RvD1 reduces nociceptor excitability, measured as decreased repetitive firing during TTX-resistant spiking. This effect is attenuated by Nav1.8-directed pharmacology or loss-of-function, supporting a Nav1.8-dependent mechanism. Together, these findings support that resolvins and related SPMs shorten the duration of immune-to-neuron sensitization and reduce the likelihood that acute inflammation consolidates into chronic pain [167]. IL-10 is a pro-resolving cytokine that can act directly on sensory neurons. In a cisplatin neuropathic pain model, IL-10 deficiency or sensory neuron-specific IL-10R1 deletion delays pain resolution, and IL-10 suppresses cisplatin-induced spontaneous DRG activity, supporting reduced neuronal hyperexcitability [168]. Organ-specific deployment of these pathways is detailed in Section 5.

### 4.3. Neuroimmune Feedback Loops and Reflex Circuits

The bidirectional nature of sensory neuroimmune communication is most clearly manifested in the formation of self-reinforcing feedback loops that coordinate neuronal activation, immune responses, and effector behaviors. The following examples illustrate how sensory input, central processing, and effector behaviors feedback to modify barrier immune states.

#### 4.3.1. Itch–Scratch Cycle

Peripheral initiation of itch depends on activation of pruriceptors and on immune-state–dependent priming that lowers the threshold for subsequent pruritogenic inputs. As discussed in Section 4, epithelial alarmins and type 2 cytokines in barrier tissues can function as sensitizers rather than obligate acute pruritogens. In a dry-skin model, conditional deletion of sensory neuron ST2, also termed IL1RL1, attenuated chronic itch, supporting a neuron-intrinsic role for IL-33 signaling in barrier-associated pruritus [156]. In human DRG cultures, brief exposure to IL-4, IL-13, or IL-33 increased Ca^2+^ responses to histaminergic and non-histaminergic pruritogens and induced transcriptional changes, supporting priming of sensory neurons by type 2 cytokines [10]. Evidence supports that Calcrl and Lbx1 spinal projection neurons are required for mechanical itch-evoked scratching and that mechanical and chemical itch engage distinct spinoparabrachial pathways targeting different FoxP2 parabrachial neuron populations. These pathways can converge in pathological itch, increasing scratching when peripheral sensitization enhances both mechanical and chemical itch signaling [169]. Scratching can disrupt the epidermal barrier and promote nociceptor-driven neuropeptide release, thereby modifying local inflammation. In mice, scratching enhanced FcεRI-dependent mast cell activation via substance P signaling through MRGPRB2, increasing cutaneous inflammation while improving defense against superficial Staphylococcus aureus infection [170]. In parallel, itch-associated cytokine pathways can include negative-feedback elements that limit inflammation. Prior work has demonstrated that IL-31-responsive sensory neurons mediated IL-31–dependent neurogenic inflammation that restrained allergic skin inflammation, supporting a neuron-driven immunomodulatory mechanism within an itch-associated cytokine circuit [171]. At the level of sensory neuron competence, a 2023 Cell Reports study demonstrated that sensory neuronal STAT3 is required for constitutive expression of IL-31 receptor genes and for IL-31–induced itch, and that sensory neuronal STAT3 also contributes to inflammatory itch in dermatitis models [11].

#### 4.3.2. Cough Reflex

In the airway, similar neuroimmune logic is implemented through vagal afferent pathways that initiate cough as a barrier-defense reflex. Cough is initiated when airway-innervating vagal afferents detect tussive chemicals, aeroallergens, or inflammatory mediators released during infection or allergic inflammation. Many triggers converge on chemosensitive vagal afferents, and inflammation can increase their responsiveness. In asthma-relevant models, IL-13 reprograms lung-innervating sensory neuron subsets, increasing defensive airway reflex sensitivity [12]. Cough-like behavior is integrated by brainstem circuits that receive synaptic input from airway vagal afferents. Tac1 neurons in the nucleus of the solitary tract receive bronchopulmonary chemosensory and mechanosensory inputs and are required for tussigen-evoked defensive expulsion [172]. It has been reported that epithelial chemosensory cells in the hypopharynx and larynx form channel synapses with vagal nerves and drive airway protective expulsion. Aeroallergen exposure increases the responsiveness of this epithelial-to-vagal pathway, indicating that allergic inflammation can enhance reflex gain [173]. Cough alters exposure and clearance at airway barriers by expelling irritants and pathogens, modifying mucus transport, and changing mechanical stress that influences epithelial signaling. Recurrent activation can drive cough hypersensitivity through increased vagal afferent excitability and central gain. Clinical benefit of P2X3 antagonism supports a role for neuropeptide-driven activation of vagal sensory pathways [174].

#### 4.3.3. Peristalsis

Enteric nervous system circuits coordinate excitatory and inhibitory motor programs to generate peristalsis and segmentation for propulsion and mixing. Inflammation can shift these programs toward hypomotility or hypermotility by acting on enteric neurons, enteric glia, smooth muscle, and immune cells, altering propulsion efficacy and contractile patterning [175]. Mechanosensory transduction within enteric circuits provides a direct route by which luminal mechanical forces regulate motility and intersect with immune homeostasis. A recent study reported that Piezo1 expressed by cholinergic enteric neurons is required for pressure sensing that supports colonic motility and peristalsis; loss of Piezo1 in this neuronal compartment reduced cholinergic neuronal activity, slowed propulsion, and was linked to impaired control of intestinal inflammation in colitis-relevant settings [176]. Inflammation can also impair motility by directly damaging enteric neuronal networks through immune–enteric nervous system interactions. In a postoperative ileus model, investigators found that surgical trauma induces early myenteric neuronal activation followed by transcriptional and translational signatures consistent with neuronal death and synaptic injury, alongside macrophage programs that create a neurodegenerative tissue environment and correlate with motility impairment [177]. These findings support an immune-to-enteric mechanism by which acute inflammation disrupts propulsion. Peristalsis also shapes barrier immunity by regulating microbial distribution, mucus organization, and mucosal antigen exposure. Experimental changes in transit time alter fecal microbiome composition and bile acid profiles, supporting a causal link between motility and microbial ecology [178]. Reduced propulsion prolongs luminal exposure to inflammatory mediators and microbial products, whereas accelerated transit alters nutrient availability and microbial growth, with downstream effects on mucosal immune tone. Organ- and compartment-specific mechanisms are addressed in the organ-specific sections.

## 5. Organ-Specific Sensory Neuroimmune Circuits

### 5.1. Neuroimmune Interactions That Regulate Barrier Function

Sensory neurons are found in all mucosal and epithelial barrier tissues, such as the skin, lungs, and gastrointestinal tract. They detect stimuli such as physical damage, temperature fluctuations, and pathogen-associated molecular patterns (PAMPs). They promptly relay this information to nearby immune cell populations, thereby creating an anatomical foundation for bidirectional neuroimmune crosstalk [2,96]. These interactions between the nervous and immune systems facilitate rapid identification of pathogens, allergens, and tissue injury, and together they are crucial for regulating tissue immunity and maintaining tissue homeostasis [15]. This section explores sensory–neuroimmune circuits specific to organs, with an emphasis on how unique anatomical, cellular, and molecular characteristics influence localized host defense, inflammation, and the maintenance of tissue homeostasis.

### 5.2. Sensory Neuron Regulation of Skin Immunity

The skin is the body’s largest primary interface with the external environment and relies on its structural integrity to maintain homeostasis, ensure practical barrier function, and defend against invading pathogens. Skin-innervating sensory neurons are closely associated with structural and functional cell types, including keratinocytes, fibroblasts, endothelial cells, Schwann cells, and resident immune cells [6,179]. These neurons are classified into three categories: Aβ-fibers, Aδ-fibers, and unmyelinated C-fibers. Among these, C-fibers (which include nociceptors and pruriceptors) have a much slower conduction velocity compared to the other two types, end within the epidermis, and form the closest links with immune cells, such as mast cells, macrophages, dendritic cells (DCs), γδ T cells, CD^4+^ T cells, and ILCs [6,180]. Additionally, these C-fibers, together with other sensory afferents, express significant TRP ion channels, especially TRPV1 and TRPA1. These channels act as molecular detectors for thermal stimuli, inflammatory factors, and pathogen-associated molecular patterns, thereby connecting sensory detection to immune regulation [1,3].

Furthermore, sensory neurons in the skin are classified into peptidergic (PEP) and non-peptidergic (NP) populations [181]. Despite this classification, both neuronal subsets express neuropeptides that modulate immune cell function. Studies show that PEP neurons produce Substance P and CGRP. In contrast, NP neurons also express CGRP, along with other neuropeptides, including TAFA4 and somatostatin (SST) [181,182].

Thus, this configuration enables rapid neuroimmune interaction, which is crucial for unified barrier defense. Consequently, we will now address how the nervous system influences immune responses in different skin disorders [183].

### 5.3. Itch and Atopic Dermatitis

Atopic dermatitis (AD) is a long-standing inflammatory skin disorder in which Chronic pruritus is a cardinal symptom, arising from complex interactions among pro-inflammatory cytokines, keratinocytes, and histamine-independent C fibers [184]. In fact, pruritus is triggered when non-histaminergic pruritoreceptors are activated by exogenous and endogenous stimuli (pruritogens), including allergens, keratinocyte-derived proteins, mast cell factors, pathogen-derived molecules, and cytokines. In atopic dermatitis (AD) lesions, skin hyperinnervation is primarily driven by a profound dysregulation and imbalance between nerve elongation factors, such as nerve growth factor (NGF), and repulsion factors, such as semaphorin 3A (Sema3A), produced by keratinocytes [185]. In normal skin, Sema3A is more active and limits the penetration of nerve fibers into the epidermis. In AD, Sema3A levels are reduced, whereas NGF levels are elevated, resulting in increased TRPV1 expression on cutaneous nerve endings. In addition, NGF upregulates the expression of substance P and CGRP, thereby promoting mast cell activation, increased itch sensitivity, and neurogenic inflammation [186,187].

Furthermore, in response to barrier disruption, proteases, and inflammatory cytokines (including IL-4, IL-13, IL-31, and IL-33), keratinocyte-derived TSLP can directly activate pruritoceptive TRPV1^+^ TRPA1^+^ neurons and promote feed-forward loops in AD-associated itch. This direct epithelial-neuronal communication may explain why AD patients experience itch before visible eczematous lesions, before substantial immune cell recruitment occurs [184,185].

### 5.4. Psoriasis

Psoriasis is an inflammatory skin disorder characterized by hyperproliferative keratinocytes, neutrophilic microabscesses in the epidermis, increased dermal angiogenesis, and immune cell infiltration. Notably, the epidermis of psoriatic lesions exhibits enhanced C-fiber innervation, and clinical observations demonstrate that 60–90% of psoriasis patients experience itching, skin pain, and discomfort, symptoms linked to abnormal activation of TRPV1 and TRPA1 channels on sensory neurons. In fact, TRPA1 activation plays a pivotal role in upregulating the expression of inflammatory cytokines, including IL-4, IL-1β, IL-16, the substrate P (SP), and NGF, thereby resulting in chronic itch and psoriasiform dermatitis (PsD) [188,189].

Mechanistic studies using imiquimod (IMQ)-induced psoriasis models have elucidated the critical role of nociceptive sensory neurons in disease pathogenesis. IMQ application induces a transient increase in CGRP expression in DRG within 6 h, followed by neuronal CGRP secretion into the skin. This neuron-derived CGRP drives IL-23 production by dendritic cells, which, in turn, stimulates γδ T cells to produce IL-17A, establishing the TRPV1^+^ sensory neuron/CGRP/IL-23/γδ T cell axis that promotes psoriatic inflammation [181,190,191].

Furthermore, a recent study showed that, in an IMQ-induced psoriasiform dermatitis model, resiniferation (RTX)-mediated sensory denervation suppressed the Type 17 immune response by markedly decreasing initial IL-23 expression, attributable to the near-complete absence of neuron-derived CGRP [192]. SP also contributes to psoriasis pathogenesis through direct effects on dendritic cells, promoting their maturation and IL-12p70 and IL-23 secretion via activation of the neurokinin-1 (NK-1) receptor and NF-κB signaling [193].

### 5.5. Bacterial, Viral and Fungal Defense

Infections caused by bacteria, viruses, and fungi are often linked with pain. Recent studies have shown that nociceptor neurons are actively involved in the skin’s antimicrobial defense. Key immunostimulatory signals produced by bacteria and fungi consist of LPS, flagellin, bacterial toxins, and zymosan. These substances modulate the functions of macrophages, dendritic cells, T cells, and innate lymphoid cells, thereby influencing immune responses and disease outcomes [194]. Recent research indicates that bacterial products, such as N-formylated peptides and LPS, stimulate somatic and visceral nociceptor neurons by engaging TRPA1 channels and Toll-like receptors (TLRs), thereby enabling rapid neural detection of bacterial invasion [195].

In staphylococcal skin infections, TRPV1^+^ neurons predominantly exert immunosuppressive effects via neurotransmitters, including CGRP [196]. In fact, CGRP primarily exerts its biological effects by binding to the calcitonin receptor-like receptor (CLR) in combination with receptor activity-modifying protein 1 (RAMP1), thereby inhibiting neutrophil recruitment to infection sites, promoting macrophage polarization toward anti-inflammatory M2 phenotypes, and suppressing inflammatory M1 macrophage activation [195].

Conversely, TRPV1^+^ neurons can mediate protective antiviral responses following skin injury. TRPV1 signaling is critical in promoting IL-27 production by myeloid cells, which subsequently stimulates keratinocytes to produce the antiviral protein (AVP) [8]. Genetic knockout of TRPV1 or RTX-induced ablation impairs wound-induced AVP responses and increases susceptibility to herpes simplex virus (HSV) infection in skin explants. This TRPV1-mediated antiviral circuit operates rapidly, providing immediate innate defense against viral pathogens at barrier sites [8].

Alongside bacteria and viruses, fungi that cause disease also trigger sensory neuron–immune pathways that generally promote inflammation and host defense mechanisms. Recent research suggests that nociceptor neurons, especially TRPV1^+^ and TRPA1^+^ fibers, react to components of the fungal cell wall, such as β-glucans and zymosan. Stimulation of these neurons triggers a localized type 17 immune response, initiated by IL-23 production by dendritic cells and macrophages, followed by IL-17 release from γδ T cells and innate lymphoid cells. This sequence results in strong neutrophil recruitment and effective management of fungal pathogens at epithelial surfaces. Activating TRPV1^+^ neurons via optogenetic or chemogenetic methods is sufficient to induce type 17 inflammation, characterized by elevated IL-6, IL-23, and TNFα levels, along with γδ T cell activation, thereby protecting β-glucan derived from Candida albicans [197].

Recent findings have added an essential layer to this model by showing that nociceptor neurons also restrain immune pathways that are poorly suited for fungal clearance. Studies demonstrate that TRPV1^+^ neurons limit the expansion and activation of innate lymphoid cell type 2 (ILC2s) during candidiasis. When nociceptor signaling is disrupted, ILC2s produce excessive amounts of type 2 cytokines such as IL-5 and IL-13, which dampen protective IL-17–mediated responses and impair fungal control. By releasing neuropeptides such as CGRP, sensory neurons act as a checkpoint that prevents inappropriate immune polarization and helps maintain a balanced antifungal response [197,198].

Interestingly, the immune response triggered by nociceptor activation during fungal infections is fundamentally different from that observed in bacterial infections. Whereas neuropeptides such as CGRP typically reduce inflammation during bacterial skin infections, the same neuronal pathways can enhance inflammatory signaling in response to fungal challenges. These opposing effects are likely due to variations in how pathogens are recognized, context-specific release patterns of neuropeptides, and differences in receptor expression profiles on immune cells [114,199]. Consistent with this, disruption of sensory neuron signalling blunts IL-17-dependent neutrophil responses and increases susceptibility to fungal infection, underscoring the essential role of nociceptors in antifungal immunity [15].

Together, these studies highlight the skin as a prime example of how nociceptor neurons act as vigilant immune sentinels that both initiate and restrain inflammation depending on pathogen context. Similar strategies are at work in other barrier tissues, such as the lungs, where sensory neurons continuously interpret environmental cues and fine-tune immune responses, helping the body defend itself while protecting tissues from excessive damage.

### 5.6. Sensory Neuron Regulation of Lung Immunity

The process of respiration continually exposes the lungs to various harmful external agents, including airborne pollutants, pathogens, and other forms of injury. Consequently, identifying these harmful threats can enhance several defense mechanisms, including immune signaling and neuronal circuit activation. The vagus nerve innervates the respiratory system [200]. Vagal sensory neurons, which are located in two separate ganglia known as the nodose and jugular ganglia, provide the primary sensory input to the airways. These sensory afferents directly respond to cytokines (IL-1β, TNF, IL-5), histamine, prostaglandins, and leukotrienes, enabling them to detect and recognize inflammatory states [201].

In the lung, pulmonary neuroendocrine cells (PNECs) are the only innervated epithelial cells and play a critical role in maintaining lung hemostasis, including respiratory regulation, immune defense, vascular control, and the recognition of mechanical stress. PNECs cluster into neuroepithelial bodies (NEBs) along the airways, which serve as reservoirs of stem cells and contain dense-core vesicles filled with bioactive neuropeptides, including CGRP, serotonin, and gastrin-releasing peptide (GRP) [202]. Recent findings indicate that PNECs express roundabout receptors (Robo), which regulate their clustering. Consequently, genetic disruption of Robo signaling impairs PNEC clustering and NEB formation, and increases neuropeptide production upon air exposure [202,203].

### 5.7. Asthma and Allergic Airway Inflammation

The development of asthma involves essential interactions between the nervous and immune systems and is characterized by airway inflammation and heightened airway sensitivity. Pulmonary neuroendocrine cells (PNECs) are found in the airway epithelium, near the branching points of the airways, which positions them close to ILC2s and thereby promotes local immune activation [204]. ILC2s are activated by alarmin released from epithelial cells and selectively express the neuromedin U receptor 1 (NMUR1). In contrast, cholinergic neurons in the enteric system and pulmonary neurons produce the neuropeptide neuromedin U (NMU). Recent research has demonstrated that stimulating ILC2s with NMU, both in vitro and in vivo, induces immediate cell activation, proliferation, and the release of type 2 cytokines, including IL-5, IL-9, and IL-13, as well as amphiregulin and GM-CSF. This occurs through NMUR1-dependent pathways that involve ERK1/2 phosphorylation and calcium/calcineurin/NFAT signaling [205,206]. Notably, NMU induces cytokine expression with more rapid kinetics than the canonical ILC2-activating cytokines, such as IL-33 and IL-25, suggesting that neuronal-ILC2 circuits function as precocious threat sensors that are activated before overt tissue damage [207].

In airways exposed to allergens, NMU acts in concert with epithelial alarmins to enhance ILC2-driven inflammatory responses. The simultaneous administration of NMU with IL-25 or IL-33 significantly worsens allergic lung inflammation, airway hyperreactivity, eosinophilia, and goblet cell hyperplasia compared with either stimulus alone [206]. Research involving humans shows that the number of NMUR1+ ILC2 cells in sputum significantly increases within 7 h after allergen exposure in individuals with mild asthma, with most NMUR1+ ILC2 Cells producing IL-5 and IL-13. The frequency of NMUR1+ ILC2s is inversely related to the dosage of inhaled corticosteroids, indicating that these neuro-activated ILC2s may act as early responders that are sensitive to corticosteroid treatment [206,208].

Conversely, several neural pathways dampen ILC2-mediated airway inflammation. β2-adrenergic receptor (ADRB2) signaling acts as a negative regulator of ILC2 proliferation in the lungs and intestines by activating the sympathetic nervous system. The α7 nicotinic acetylcholine receptor (α7nAChR), which mediates rapid excitatory synaptic transmission, has emerged as a potential therapeutic target in neuropsychiatric, neurodegenerative, and inflammatory diseases by inhibiting NF-κB signalling pathways [209,210]. Moreover, CGRP exerts complex, context-dependent regulatory effects on ILC2 responses. While CGRP produced by pulmonary neuroendocrine cells (PNECs) may contribute to asthmatic inflammation, CGRP derived from activated ILC2s can act as a negative feedback signal to suppress inflammatory responses [208].

Neuromedin B (NMB) functions as an endogenous brake on ILC2 responses [211]. Sensory neurons release neuropeptide NMB in response to helminth infections and allergic inflammation to suppress ILC2 activation, eosinophil accumulation, and mucus secretion. Prostaglandin E2 (PGE2) produced by basophils enhances this feedback loop by increasing NMB receptor (NMBR) expression on ILC2s [212]. Adenosine, produced from extracellular ATP during epithelial barrier challenges, also inhibits ILC2 responses by activating the A2A receptor and inducing cAMP-mediated suppression of NF-κB [208,212].

### 5.8. Viral Defense

The trachea, bronchi, and airways are innervated by peripheral sensory afferents that play critical protective roles during respiratory viral infections and help balance antiviral immunity. During influenza A virus (IAV) infection, vagal TRPV1^+^ sensory neurons provide essential host protection. From a mechanistic perspective, the removal of TRPV1^+^ neurons during influenza A virus (IAV) infection leads to improper recruitment of myeloid cells, resulting in significant elevations of lung monocytes, neutrophils, and inflammatory macrophages derived from monocytes [213,214]. The increased mortality in nociceptor-deficient mice results from exaggerated immunopathology rather than uncontrolled viral replication. Recent studies have shown that depletion of myeloid cells improves survival in RTX-treated, IAV-infected mice, confirming that excessive myeloid recruitment underlies the fatal immunopathology. These findings reveal that TRPV1^+^ sensory neurons and CGRP administration limit viral spread while simultaneously constraining potentially fatal inflammatory responses [8].

Simultaneously, acetylcholine (ACh) produced by B cells acts as an additional neuroimmune regulatory mechanism during viral infections affecting the respiratory system. B cells that generate ACh are identified as early modulators of lung inflammation. When choline acetyltransferase (ChAT) is genetically disrupted in these B cells, this disruption alters the activation of interstitial macrophages (IMs) [215]. ACh inhibits TNF production by immune cells during the initial stages of IAV infection, thereby reducing viral control while also averting excessive inflammatory reactions. Mice with a B cell-specific knockout of ChAT exhibit lower early viral loads but later experience heightened local and systemic inflammation, along with impaired epithelial repair at subsequent time points, even though viral clearance remains comparable [192].

Additionally, initial innate immune responses are essential for controlling viral spread. Prompt and efficient interferon responses limit viral replication, reduce viral-induced inflammation, and help avert progression to severe illness. Delayed or insufficient interferon responses result in persistent viral replication that drives excessive inflammatory reactions and contributes to severe morbidity and mortality. Collectively, these observations indicate that neuroimmune interactions modulate critical early interferon responses through multiple pathways [216,217].

### 5.9. Sensory Neuron Regulation of Gut Immunity

The gastrointestinal tract contains the enteric nervous system (ENS), a vast neuronal network of 200–600 million neurons structurally arranged into myenteric and submucosal plexi [218,219]. The ENS operates in a partially independent manner and therefore maintains bidirectional communication with the central nervous system via extrinsic sensory neurons located in the DRG and the vagal ganglia. One of the most essential interactions is between the intrinsic primary afferent neurons (IPANs) in the ENS. These neurons directly contact both enteric interneurons and motor neurons, forming a reflex circuit that controls various aspects of digestive function [218,220].

Gastrointestinal sensory neurons have a diverse set of molecular sensors, such as TRP channels (TRPV1, TRPA1, TRPM8), receptors for neuropeptides, cytokines (IL-1β, TNF, IL-17), as well as pattern recognition receptors, including TLRs. This molecular arsenal of intestinal sensory neurons enables them to recognize bacterial products, inflammatory mediators, and tissue damage [221]. In response to the stimulus, sensory neurons also induce the secretion of neuropeptides, including substance P, CGRP, VIP, and PACAP, which, in turn, regulate the immune response [221,222].

### 5.10. Inflammatory Bowel Disease

IBD is a model of chronic intestinal inflammation that is associated with dysregulated neuroimmune interactions. Inflammatory bowel disease (IBD) patients manifest enhanced abdominal pain, which also includes a burning sensation. This symptom is associated with increased expression of TRPV1^+^ immunoreactive fibers in inflamed intestinal tissue [223]. Experimental models of colitis show that TRPV1 antagonists reduce colon shrinkage, histological damage scores, and weight loss. In contrast, the activation of TRPV1 aggravates the increase in mechanical and thermal pain sensitivity during gastrointestinal inflammation [224].

Neuropeptides have intricate effects on intestinal inflammation. One of the substances in this group, Substance P, acts through neurokinin receptors (NK1R, NK2R, NK3R), which are expressed on neurons and immune cells. Inflammatory bowel diseases are characterized by increased SP, which correlates positively with disease activity, whereas NK-1 receptor expression is upregulated in intestinal vessels and lymphoid structures. SP increases both mast cell activation and degranulation. These mast cells become the executors of neurogenic inflammation. On the other hand, SP is capable of triggering anti-inflammatory effects as well by NK1R signal transduction, which blocks macrophage infiltration and elevates M2-like macrophage polarization levels [83,225].

CGRP is a substance that, in most cases, helps to reduce the inflammatory response in the gut. Inflammatory bowel disease (IBD) is one of the conditions in which lowered levels of CGRP have been observed. Activation of TRPM8, which leads to CGRP release, has been shown to reduce the severity of TNBS-induced colitis, as evidenced by reduced histological damage, bowel thickness, myeloperoxidase activity, and pro-inflammatory cytokine production [226]. CGRP shifts macrophage polarization toward M2 cells while preventing M1 activation, decreasing the production of TNFα, IL-1β, and iNOS, and increasing IL-10 expression. CGRP uses cAMP-dependent pathways to reduce both NF-κB activation and pro-inflammatory cytokine transcription in macrophages [227].

VIP is a key immunoregulatory neuropeptide of the gut with well-known anti-inflammatory effects. VIP is a key player in immunological tolerance and homeostasis, mainly functioning through the VPAC2 receptor. This receptor inhibits Th1 and Th17 activities while inducing Th2 and regulatory T cell differentiation [228]. VIP treatment in experimental rat models of colitis alleviates disease symptoms, reduces mortality from septic shock, and mitigates detrimental effects in rheumatoid arthritis models by downregulating inflammatory and autoimmune responses. VIP blocks the production of TNFα, IL-6, IL-12, and iNOS, while promoting IL-10 expression, in the activated macrophages and microglia [229].

Gut immune responses are controlled by sensory neurons, as shown in chemogenetic and optogenetic activation studies. One of the significant changes in immunomodulation in the colon following activation of TRPV1^+^ spinal sensory neurons is a reduction in RORγ^+^ regulatory T cells (Tregs), along with changes in innate lymphoid populations and macrophages [13]. TRPV1^+^ DRG neurons reduce colonic Treg cells by release of CGRP that acts on RAMP1 receptors [230]. Cholinergic neurons control the functions of neutrophils, whereas Nos1^+^ neurons modulate Th17-like cells, thereby showing that different neuronal subsets are particular in their regulation of specific immune cell populations [231].

### 5.11. Parasite Expulsion

Helminth infections activate neuroimmune responses, which are necessary for the removal of the parasite. Mucosal ILC2s are the primary mediators of the immune response against helminths and are a significant source of IL-13, which targets intestinal epithelial and goblet cells. This Process eventually leads to hyperplasia, increased mucus production, and increased muscle contractility, known as the “weep and sweep” response, which allows worm expulsion [232].

Tuft cells, one of the rare types of chemosensory epithelial cells known for their expression of bitter taste receptors, have been shown to powerfully influence anti-helminth type 2 responses via their role as the primary source of IL-25 in the epithelial mucosa and lamina propria. These cells are the dominant source of IL-25, maintaining ILC2s in the tissues of the lower gastrointestinal tract in a resting state [125]. In the detection of helminth infection, cells sense the arrival of a parasite-derived product via their G-protein-coupled taste receptors and thereby activate phospholipase Cβ2 (PLCβ2) through signaling cascades, which ultimately lead to the secretion of IL-25. The IL-25 released from the tuft cells is the signal that the immune cells called ILC2s need to produce the cytokine IL-13 which then morphs the epithelial crypt progenitors to the extent that the production of new tuft and goblet cells is initiated; thus, an increase in cell number of this type can be considered the result of a feed-forward amplification circuit [233,234].

Enteric neurons can directly detect helminth products and, in response to parasitic infection, release NMU. Intestinal ILC2s are found in proximity to cholinergic neurons that produce NMU. Activation of NMUR1 on ILC2s results in a rapid increase in cell numbers and in the production of IL-5, IL-9, and IL-13. Providing an external supply of NMU to a mouse infected with Nippostrongylus brasiliensis facilitates clearance of the parasite from the body [232]. On the other hand, mice lacking NMU or NMUR1 exhibit increased worm burdens and impaired anti-helminth immune responses. The neuronal-ILC2 reaction is much faster than those triggered by IL-33 or IL-25, thus enteric neurons are likely to be the first cells to detect a helminth attack [233].

During helminth infections, VIP released from enteric neurons in response to feeding is another neural signal that regulates ILC2 function. ILC2s receive VIP signals via VIP receptor 2 (VIPR2) to increase IL-5 production and eosinophil recruitment. IL-5 acts directly on nociceptors to accelerate VIP release, thereby establishing a feed-forward loop that amplifies type 2 inflammation [221,235]. On the other hand, epinephrine discharged from the sympathetic nervous system through β2-adrenergic receptor (β2AR) signaling inhibits ILC2 responses, hence, a mechanism to prevent ILC2 overactivation and possible immunopathology [13].

ILC2s are the ones that produce acetylcholine themselves, which serves as an autocrine function to maintain ILC2 groups in the event of a helminth infection [236]. Impairment of the choline acetyltransferase gene exclusively in ILC2s leads to increased parasite numbers, reduced ILC2 numbers, and weakened lung and intestinal barrier responses during N. Brasiliensis infection. The ILC2-intrinsic cholinergic signaling is one fascinating way for ILC2s to control their own proliferation to the greatest extent; type 2 immunity has been generated [237].

### 5.12. Microbiota-Neuron Interaction

The gut microbiota is a significant factor that determines the enteric nervous system (ENS) development, maturation, and function during the whole lifespan. Germ-free (GF) mice have an immature ENS, and their neuroanatomy is different; also, the neuronal subtypes have changed in proportion, and the intestinal transit is slowed when these mice are compared to conventionally raised (CONV-R) mice. The reestablishment of the microbiota in adult GF mice returns the ENS morphology and function to the normal level within 15 days; this is strong evidence that the microbiota is a factor that can still induce ENS maturation even in adulthood [238].

Microbiota-ENS communication is mainly mediated through serotonin (5-HT) signaling [238]. Enterochromaffin cells of the intestinal epithelium, upon microbial stimuli, produce and release 5-HT, and enteric neurons also synthesize 5-HT. Microbiota colonization increases neuronal and mucosal 5-HT production and, consequently, stimulates the proliferation of enteric neuronal progenitors expressing nestin [238]. The 5-HT4 receptor activation is indispensable for microbiota-dependent ENS maturation; administration of the selective 5-HT4 agonist prucalopride to GF mice brings back intestinal innervation and transit to the levels of conventionally colonized mice [238].

Genetic removal of peripheral 5-HT production via the knockout of tryptophan hydroxylase 1 (Tph1) demonstrates that mucosal 5-HT acts as a protective agent for the nervous system during the initial colonization. Myenteric neuron numbers in CONV-D Tph1-/- mice are reduced, and the percentage of nestin^+^ neurons is also decreased after colonization, which emphasizes that neuronal and mucosal 5-HT pools are indispensable for the maintenance of ENS structure during microbial colonization [218,238].

Microbiota also regulate ENS activities through several other pathways. Different bacterial strains affect the excitability of enteric neurons; Lactobacillus reuteri, for example, increases sensory neuron excitability by blocking calcium-dependent potassium channels, thereby altering gut motility and pain perception. Microbiota produce a variety of neuroactive compounds, including GABA, serotonin, melatonin, histamine, and acetylcholine, which can act as local neurotransmitters to influence ENS activity. The short-chain fatty acids (SCFAs) and secondary bile acids that are microbial-derived are also the primary sources of energy for enteric neurons, and thus they regulate motility [238].

Toll-like receptor 2 (TLR2) signaling connects gut bacteria with the adult Enteric Nervous System (ENS) maintenance via neurogenesis regulation. Microbiota from the colon induce neurogenesis of neural precursors from the enteric nervous system in a manner dependent on TLR2. Blocking TLR2 signal transduction impairs colonic neurogenesis, whereas TLR2 stimulation enhances it, also in antibiotic-induced dysbiosis and germ-free situations. These results reveal the mechanism by which dysbiosis can lead to dysfunction of the ENS and thus be the cause of motility disorders [239,240].

Muscularis macrophages (MMs) represent a significant cellular population that links the microbial and neural compartments of the intestinal wall to each other. MMs mature next to enteric nerve fibers and need colony-stimulating factor 1 (CSF1) produced by the surrounding enteric neurons to live and to be active. Besides that, MMs produce and release bone morphogenetic protein 2 (BMP2), a member of the TGF-β superfamily, which regulates enteric neuron function [241,242]. LPS originating from gut microbes mediates this two-way communication by regulating BMP2 production by MMs and CSF1 production by enteric neurons. The three-way interaction of microbes, macrophages, and neurons is essential for the preservation of intestinal homeostasis and for normal motility [243,244].

Research across various systems, such as the skin, lungs, and gastrointestinal tract, indicates that sensory neurons play a crucial role in barrier immunity rather than merely serving as passive channels for pain or reflex responses. By detecting disturbances in tissues and microbial signals, sensory neurons rapidly influence the recruitment, polarization, and functional responses of immune cells through context-sensitive release of neuropeptides and neurotransmitters. These pathways can enhance protective immunity—such as IL-17–mediated antifungal defense and antiviral responses—while also imposing essential limits on inflammation to prevent tissue damage. Disruption of these mechanisms leads to chronic inflammatory conditions, increased susceptibility to infections, and neuropathic symptoms, underscoring the role of sensory neurons as protectors of barrier integrity and as potential therapeutic targets.

## 6. Dysregulation in Chronic Inflammatory and Allergic Diseases

A recurring theme across barrier tissues is that sensory neurons do not simply report inflammation; they reshape it. When these circuits become persistently overactive, they can amplify pain, itch, edema, bronchospasm, mucus production, dysmotility, and shifts in immune cell positioning. When they become underactive, the same neuroimmune wiring that normally supports protection can fail, leaving tissue more vulnerable to infection, poor repair, and dysregulated inflammation.

This section links back to the mediator logic summarized earlier (Section 3) and the organ-level circuit layouts (Section 5). It also sets up the therapeutic “entry points” shown in Table 4.

### 6.1. Hyperactive Sensory Neuroimmune Circuits in Chronic Pain and Chronic Itch

Chronic pain and chronic itch share a logic that is easy to miss if symptoms are kept in separate clinical boxes. Peripheral sensory neurons (including TRPV1/TRPA1-lineage nociceptors and specialized pruriceptors such as MrgprA3-related programs in mice, with human correlates across MRGPR families) detect inflammatory mediators and tissue stress signals. Many of those mediators also modify neuronal excitability directly, so the afferent arm becomes easier to trigger and harder to silence. In parallel, activated neurons release neuropeptides and transmitters (CGRP, substance P, VIP, glutamate, ATP) that feed back onto immune and stromal cells, shifting cytokine production, vascular behavior, and leukocyte recruitment. This forms a positive feedback loop that can keep symptoms “on” even when the original trigger has faded [2,261,262].

The “neuronal sentinels” framing helps here because it forces a more direct causal question: in a given disease, is neuronal activity only reflecting immune tone, or does neuronal activity become part of what sustains the inflammatory state? The answer varies by tissue and trigger, but recent syntheses argue that sensory neurons can function as first responders that shape downstream immune programming rather than simply reporting local damage [123].

Type 2 cytokines provide a concrete example of how immune programs tune sensory gain. Human sensory neurons exposed to IL-4/IL-13/IL-31/IL-33/TSLP show increased excitability and enhanced responses to itch-evoking stimuli, connecting cytokine state to symptom intensity in mechanistic terms [261,263,264]. Clinically, the speed of itch relief seen with targeted therapies (especially IL-31 axis biologics and JAK inhibition) supports the idea that neuronal sensitization is not just downstream noise; it can drive perceived disease severity and scratching-related barrier injury [250,251].

Scratching itself adds a second layer of dysregulation. Mechanical injury disrupts the epidermal barrier, increases antigen penetration, and promotes alarmin release, which can further engage sensory endings and local immune cells. Mast cell–neuron interactions are central: mast cell mediators activate sensory terminals, sensory peptides influence mast cell behavior, and non-IgE pathways (including MRGPR-linked mast cell activation routes) can sustain flares even when classic allergen-driven mechanisms are not dominant [187,262]. In this setting, hyperactivity is not only electrical; it is circuit-level, involving cytokines, neuropeptides, epithelial stress signals, and immune activation in a closed loop.

A parallel hyperactivity pattern appears in inflammatory pain states where neuronal firing and neurogenic inflammation sustain tissue-level inflammation. CGRP and substance P can increase local blood flow and vascular permeability, shaping edema and leukocyte trafficking, and in some contexts, they regulate cytokine output from resident immune cells [2]. The direction of these effects is not uniform across organs or models. Some settings show CGRP dampening aspects of type 2 inflammation, whereas others show symptom amplification through neurovascular effects and immune modulation. This context dependence matters for translation because it predicts that blocking a sensory mediator may relieve symptoms in one tissue but carry trade-offs for host defense in another [2,265].

The itch–scratch cycle in atopic dermatitis is a clean example of a circuit dominated by type 2 cytokines and epithelial alarmins feeding into pruriceptor gain, then back into barrier injury and alarmin release [263,264]. Chronic inflammatory pain more often mixes neuropeptide-driven neurovascular effects, innate immune activation (macrophages, mast cells), and injury-related changes in ganglia and peripheral terminals [2,262]. Those differences are why symptom speed differs across targets (see also Section 6.1 on endpoint selection).

### 6.2. Airway Disease: Neuroimmune Amplification in Asthma Exacerbations and Related Phenotypes

In asthma, symptom escalation during exacerbations can be understood as a fast neuroimmune amplification problem layered on slower immune remodeling. Airway sensory neurons detect irritants, cold air, infection-related signals, and epithelial damage products. Cytokines in type 2 inflammation can also reprogram airway sensory neurons directly. A recent study showed that cytokine exposure shifts sensory neuron properties in asthma-relevant contexts, supporting a model where immune state primes the nervous system for exaggerated reflex responses [12]. This priming increases the likelihood that otherwise tolerable stimuli trigger cough, bronchospasm, and mucus hypersecretion.

Epithelial alarmins sit near the top of this cascade. TSLP is a validated therapeutic target in severe asthma, and its biology includes broad effects on immune activation pathways that can interface with neurogenic triggers during flares [266]. IL-33/ST2 signaling is positioned similarly, linking epithelial stress to immune activation and, indirectly or directly, to sensory neuron-driven symptom states. The clinical focus is usually immune outcomes (exacerbation rate, lung function), but the mechanistic framing is broader: once alarmin-driven inflammation increases sensory excitability, symptom escalation can become self-propagating in a subset of patients.

Chronic cough illustrates the same principle with a more symptom-forward endpoint. ATP released from stressed airway epithelium activates purinergic receptors on sensory neurons, promoting cough hypersensitivity. P2X3 antagonism has become proof-of-mechanism in refractory chronic cough, showing that targeting sensory transduction can reduce symptom burden even when underlying inflammatory drivers are heterogeneous [174,253,267].

The flip side (and this links forward to Section 6.4) is that airway sensory function is not purely pathogenic. Protective reflexes (cough, bronchoconstriction to limit exposure, local neurogenic support of epithelial repair) exist for a reason. In patients with comorbid neuropathy or impaired afferent signaling, blunting sensory signaling further could plausibly worsen defense against aspiration or infection, even if symptoms improve. That is not a reason to avoid sensory targets; it is a reason to stratify and to build host defense logic into safety monitoring (expanded in Section 6.2).

### 6.3. Gut Inflammation: Neuroimmune Dysregulation in IBD Flares and Visceral Symptoms

IBD is often framed as immune dysregulation plus epithelial barrier disruption, but sensory neuroimmune circuits provide an additional lens that helps explain episodic flares, visceral pain, urgency, and the mismatch that can occur between endoscopic inflammation and symptom load. Extrinsic sensory neurons innervating the gut detect inflammatory mediators and microbial products and can influence immune behavior through neuropeptides and transmitter release. Recent syntheses consolidate bidirectional signaling between extrinsic neurons and innate lymphoid cells, macrophages, mast cells, and epithelial programs in the intestine [2,268].

Newer gut work is also sharpening which neuronal products matter and when they appear to protect versus aggravate. A notable 2025 report describes a CGRP-related peptide, adrenomedullin 2 (ADM2), as a neuron-linked signal that supports tissue-protective ILC2 responses and limits intestinal inflammation, positioning at least part of “sensory neuropeptide biology” as protective in defined contexts [269]. Therefore, CGRP-family mediators have context-dependent roles, and the manuscript should state that plainly rather than implying a single-direction effect across organs.

Tuft cell–ILC2 circuitry adds a second gut-specific layer because it ties luminal sensing and epithelial programs to downstream type 2 responses that can become dysregulated. A 2025 Nature Immunology study reported tuft cell–intrinsic IL-17RB as a regulator that restrains IL-25 bioavailability and reveals context-dependent ILC2 hypoproliferation, reframing IL-25 circuit control as more than “upstream activation” [125]. Mechanistically, this matters for IBD-like inflammation and post-infectious or microbiota-linked dysmotility phenotypes because it suggests that the same epithelial–immune loop can flip between exaggerated activation and dysfunctional hyporesponsiveness depending on where the regulatory break sits.

A strong “mapping” example comes from a chemogenetic screen implicating TRPV1-expressing afferents in shaping gut immune outcomes and suggesting that dysregulation of this crosstalk can contribute to gastrointestinal disorders that mix visceral pain and inflammatory features [13]. This type of work matters because it ties defined neuronal populations to immune consequences rather than treating “the nervous system” as a generic modifier.

During IBD flares, hyperactive circuits can present as heightened visceral pain and altered motility, but the underlying biology can also include neuropeptide-driven changes in vascular dynamics and leukocyte trafficking in the mucosa. The same neuroimmune signaling that supports rapid defense against enteric threats can, when persistent or mis-timed, sustain inflammatory recruitment and tissue stress. Microbiota-linked signals complicate the picture further because microbial metabolites and epithelial responses can tune sensory thresholds indirectly. Recent syntheses place the gut microbiota within a gut–brain–immune axis that maintains or disrupts this balance [270,271].

The emerging concept of “sensory nerve niches” defines discrete microenvironments that organize neuroimmune interactions at barrier tissues. In this framework, barrier-derived alarmins drive neuronal activation and neuropeptide release, initiating immune recruitment and cytokine feedback, as depicted in Figure 1 [272].

### 6.4. Hypoactive Circuits: Impaired Host Defense and Repair in Neuropathy-Associated States

Not all clinically important neuroimmune dysregulation is hyperactivity. In some settings the problem is insufficient sensory signaling and reduced neurogenic support for protection and repair. Diabetic peripheral neuropathy is a major example because it combines loss of protective sensation (leading to unrecognized injury) with altered local neuropeptide signaling that can impair vasodilation, leukocyte recruitment patterns, and tissue repair dynamics. Reviews of diabetic wound healing highlight impaired cutaneous innervation as a contributor to delayed healing and increased complication risk, integrating neuronal deficits with immune and vascular dysfunction [273]. A more general framing is that reduced neurogenic inflammation can blunt appropriate acute inflammatory responses after injury, impairing early defense and repair orchestration. That creates conditions favorable for infection, chronic ulcers, and prolonged inflammation.

Mechanistically, loss or dysfunction of sensory fibers can reduce local release of CGRP and substance P, signals that participate in vasodilation and immune modulation. In diabetes, this intersects with microvascular disease and immune impairment, compounding infection susceptibility. Contemporary reviews of diabetic peripheral neuropathy emphasize neurogenic inflammation concepts and broader pathophysiologic shifts after nerve injury [274]. The clinical expression is familiar—foot ulcers, recurrent infections, delayed healing—but the neuroimmune circuitry perspective clarifies why immune support can be locally defective even when systemic inflammatory markers are not extreme.

This hypoactivity logic also generalizes beyond diabetes. In the airway, reduced afferent signaling can mean weaker protective reflexes and altered local inflammatory calibration. In the gut, autonomic and sensory dysfunction can contribute to dysmotility, altered barrier behavior, and vulnerability to secondary infection or persistent inflammation after an insult. The point is not to claim one mechanism for all “low-signal” states; it is to state that host defense and repair are part of what sensory pathways do, and suppressing these pathways in the wrong subgroup could worsen outcomes (this becomes a concrete safety argument when discussing CGRP-family blockade or upstream alarmin blockade in Section 6.4).

Long COVID is currently the most visible clinical example because multiple cohorts and reviews describe small fiber neuropathy features after SARS-CoV-2 infection and suggest immune-associated mechanisms in at least a subset [275,276,277,278]. In practical terms, this means some patients present with pain, dysesthesia, dysautonomia, or altered sensory thresholds in ways that look less like “hyperactive itch/pain loops” and more like disordered signaling, sometimes with reduced protective function plus aberrant symptom generation.

This matters for a sensory neuroimmune review because it forces a cleaner separation between (i) symptoms driven by excess excitability in intact circuits and (ii) symptoms emerging from injured or maladaptive circuits where signaling is unreliable. The therapeutic implications differ. A therapy that reduces excitability may help subgroup (i) quickly, but in subgroup (ii) the same therapy could flatten already weak protective signaling or worsen functional outcomes. So, “post-viral dysregulation” belongs here not as a tangent, but as a reminder that circuit state is a variable that should be measured, not assumed.

### 6.5. Emerging Links to Systemic Diseases: Migraine, Long COVID, and Cancer

Migraine is already a translational success story for sensory neuroimmune biology, anchored in CGRP signaling. CGRP-targeted therapies validate that blocking a sensory-neuropeptide axis can reduce disease burden at scale [245,246]. That success strengthens a broader hypothesis: other systemic conditions with episodic flares or sensory-dominant symptoms may reflect neuroimmune circuit dysregulation, but with tissue-specific trade-offs.

Long COVID (post-acute sequelae of SARS-CoV-2 infection) has been linked to small fiber neuropathy and neurovascular dysregulation in a subset of patients, providing a plausible bridge between immune activation, dysautonomia, sensory symptoms, and fatigue [275,276,277,278]. This does not imply a single mechanism for long COVID. It supports including peripheral sensory neuroimmune disruption in models of ongoing symptoms when pain, dysesthesia, or dysautonomia are prominent.

Cancer adds a different systemic angle. Tumor–nerve interactions are now framed as multi-directional: sensory neurons influence tumor immunity through neuropeptides, and tumor-driven immune changes alter nervous system function and pain. Recent reviews synthesize evidence that CGRP and substance P can modulate immune behavior in tumor microenvironments, with implications for immune escape and tumor progression in some contexts [279,280,281,282]. From the standpoint of sensory neuroimmune therapeutics, this creates a tension. Blocking sensory mediators may relieve pain and shift immune tone, but the direction and magnitude of immune impact may vary by tumor type and microenvironment. It also implies that infection risk and tumor immunity endpoints should be evaluated explicitly when extending sensory-targeted drugs beyond their original indications.

## 7. Therapeutic Translation: From Anti-CGRP Success to Next-Generation Targets

Therapeutic development in the sensory neuroimmune space has moved from mechanistic curiosity to clinical reality. Anti-CGRP therapies demonstrate that a sensory-derived mediator can be targeted safely enough for widespread use with meaningful efficacy. That success has shaped expectations: investigators now ask whether other sensory nodes—ion channels, neuropeptide receptors, and cytokine-to-neuron interfaces—can be targeted with comparable specificity and tolerability.

This section should be read alongside the organ circuit framing in Section 5 and the mediator categories in Section 3. Table 5 summarizes current and emerging strategies.

### 7.1. Approved or Clinically Validated Nodes: What Worked, and What It Teaches

#### 7.1.1. CGRP Axis: Monoclonal Antibodies and Small-Molecule Antagonists

CGRP-targeted therapy is the clearest translational proof that a sensory neuropeptide pathway can be drugged successfully. Monoclonal antibodies to CGRP or its receptor (e.g., erenumab, fremanezumab, galcanezumab, eptinezumab) and small-molecule CGRP receptor antagonists (gepants) reduce migraine frequency and disability in large populations [245,246,283]. The APPRAISE randomized trial is one example supporting earlier use of erenumab relative to nonspecific oral preventives, with durable benefits at 12 months [245]. These outcomes likely reflect interruption of neurovascular and neuroinflammatory amplification that contributes to attack propensity, not simply analgesia. Nevertheless, CGRP plays important physiological roles in vascular homeostasis, and concerns remain regarding cardiovascular safety, species-specific pathway differences, and the limited efficacy of CGRP blockade outside migraine, underscoring potential constraints on broader translational applicability.

Two translation lessons stand out. First, pathway specificity matters. CGRP blockade targets a defined axis with measurable endpoints and a tolerability profile acceptable for chronic use. Second, tissue context matters. CGRP-family mediators can participate in tissue repair and host defense signaling in some settings, and recent gut work reinforces that at least some CGRP-related biology can be tissue-protective rather than uniformly pro-inflammatory [116,269] (Table 5).

#### 7.1.2. Sodium Channels: Nav1.8 as a Proof Point for Peripheral Targeting

Ion channel targeting has long been attractive, but safety and selectivity limited progress. The target zone is tightening. Suzetrigine (JOURNAVX), a Nav1.8 inhibitor, received FDA approval in 2025 for moderate to severe acute pain in adults, validating peripherally restricted sodium channel inhibition as a scalable analgesic approach [248,249]. This approval matters beyond acute pain: it supports the concept that a sensory-neuron–enriched channel can be targeted without central opioid-like effects, which is directly aligned with the “circuit node” strategy.

Nav1.7 remains genetically validated due to congenital pain insensitivity phenotypes, but translating that biology into safe and effective drugs has been difficult. Many Nav programs now emphasize selectivity, peripheral restriction, and careful cardiac safety profiling. In Table 5, Nav1.7/1.8 entries should be separated clearly: Nav1.8 now has an FDA-approved precedent (acute pain), whereas Nav1.7 remains largely investigational for chronic indications. The forward-looking question is whether peripheral sodium channel inhibition can reduce itch or neurogenic inflammation in conditions where hyperactive circuits drive disease burden, without unacceptable off-target effects.

#### 7.1.3. JAK Inhibitors: Rapid Symptom Shifts Support Neuroimmune Relevance Even When the Drug Is Framed as “Immune-Targeted”

JAK inhibitors are usually introduced as immunomodulators, not sensory therapeutics, but they provide a useful clinical signal: symptom improvement can occur quickly, including itch reduction in atopic dermatitis, implying that dampening cytokine signaling changes sensory neuron sensitization in vivo. Abrocitinib and other JAK inhibitors are approved for atopic dermatitis, and upadacitinib has approvals in ulcerative colitis and Crohn’s disease [251,284,285]. Time-course analyses in atopic dermatitis show measurable itch improvements early after treatment initiation, which is consistent with a circuit-level effect rather than only slow tissue remodeling [252]. Safety concerns (serious infection risk, thrombosis, cardiovascular events in selected populations) remain central and limit broad use, especially when safer biologics exist. Still, as a mechanistic signal, JAK inhibitors support Section 3 logic that cytokine-to-neuron signaling is a tractable symptom driver in at least a subset of patients.

### 7.2. Next-Generation Targets: Moving Toward Circuit-Selective Intervention

#### 7.2.1. IL-31 Axis: A Symptom-Forward Pathway with Clear Neuronal Relevance

IL-31 is strongly linked to itch, and IL-31 receptor expression across neural and cutaneous compartments provides a plausible bridge from cytokine programs to symptom generation. Nemolizumab (NEMLUVIO), an IL-31 receptor antagonist, received U.S. approval in 2024 for atopic dermatitis (in combination with topical therapies), and the pivotal evidence base is summarized in prescribing information [250]. This represents a case in which clinical benefit plausibly reflects direct interruption of sensory itch circuits, rather than immune disease modification alone. However, IL-31 signaling also contributes to host defense and immune regulation, and long-term receptor blockade may be limited by incomplete symptom control, compensatory activation of parallel pruritic pathways, or unanticipated immunological effects.

For translation, the key question is not whether IL-31 blockade reduces itch—it does—but which patient subgroups achieve sustained disease control versus symptom relief that unmasks ongoing inflammatory drivers requiring additional immune targeting. Table 4 comparisons help here: in patients whose disease is itch-dominant with strong neuroimmune sensitization, IL-31 axis targeting may match the dominant driver; in broader inflammatory phenotypes, it may need to sit alongside upstream anti-inflammatory control (and this is where Section 5 organ-circuit context can justify combination logic rather than making it seem ad hoc).

#### 7.2.2. IL-33/ST2 and Upstream Epithelial Programs: Benefit with Trade-Offs

Upstream epithelial alarmin targeting aims to suppress a broad cascade that includes neuroimmune amplification. Tezepelumab’s success targeting TSLP in severe asthma supports the alarmin strategy [266], and the updated label indications reinforce that epithelial-driven disease categories can be clinically tractable targets [286]. A limitation is that upstream blockade is less circuit-selective and can function as broad immunomodulation. In Table 5, IL-33/ST2 and similar upstream targets should be presented with explicit host defense and repair constraints, with the warning that circuit symptom relief is not the only endpoint that matters. This is where Section 6 “hypoactivity” logic becomes clinically relevant: suppression of upstream signals in a low-signal host defense state may carry different risks than suppression in a high-signal, symptom-dominant state.

#### 7.2.3. MRGPR-Linked Pathways: Mast Cell–Neuron Interfaces Without Classic IgE Dependence

Non-IgE mast cell activation can produce intense itch and flare behavior. MRGPRX2 is a leading target because it mediates pseudo-allergic mast cell activation and can contribute to urticaria-like phenotypes and neurogenic inflammation. Oral antagonists are in development, including programs such as EVO756 in inflammatory skin disease contexts [254]. At the same time, development challenges and safety signals have led to pauses or discontinuations in some related candidates, emphasizing that receptor selectivity and human relevance must be handled carefully [255]. Table 5 should therefore present MRGPR antagonism as an attractive circuit-interface concept with a real translation bottleneck: species differences and receptor pharmacology can turn a mechanistically clean idea into an unpredictable clinical program.

#### 7.2.4. Optogenetic-Inspired Neuronal Silencing and Gene-Enabled Circuit Control

Ion channel blockers and antibodies represent conventional pharmacology. A more radical direction is circuit control through gene-based or device-enabled neuromodulation. Preclinical optogenetic silencing of nociceptive afferents reduces pain behaviors, proving that inhibition of defined sensory inputs can modify disease-relevant outputs [259]. Reviews describe gene therapy strategies for chronic pain that include optogenetic, chemogenetic, and RNA-based approaches, with the main barriers being delivery to peripheral ganglia, long-term control, and safety [260].

#### 7.2.5. BTK Inhibition and Neuroinflammation: Plausible Relevance, but the Circuit Link Must Be Shown

BTK inhibitors are being developed and tested in neuroinflammatory diseases such as multiple sclerosis, including brain-penetrant agents intended to act on microglia and B cells [256]. This is not a sensory-axon-specific intervention. Still, BTK regulates microglial function and broader neuroinflammation in experimental models [257]. BTK also connects to inflammasome biology in innate immune cells, and inflammasome-linked pyroptosis mechanisms have been tied to neuropathic pain phenotypes in experimental systems [287,288]. The translational opportunity is that BTK inhibition might modify neuroinflammatory components that sustain chronic symptoms in conditions where immune activation and sensory dysfunction coexist. The translational risk is that this behaves like broad immune modulation with systemic safety constraints, so “neuronal inflammation” indications need mechanistic justification and endpoints beyond generic symptom scoring [258].

#### 7.2.6. Practical Translation Checkpoints for the Sensory Neuroimmune Axis

Three practical issues should guide near-term development across these targets.

First, endpoint selection must match the mechanism. Circuit-targeted therapies may change symptom intensity quickly (itch, pain, cough) even if tissue pathology changes more slowly. This is visible in cough trials that track objective cough measures and cough-related health status, and in atopic dermatitis programs that quantify itch time-course [174,252]. Trials should plan for both, with symptom endpoints tied to mechanistic biomarkers where possible: quantitative sensory testing, flare frequency, neurogenic inflammation markers, and validated patient-reported outcomes [289]. The key is to avoid the common failure mode where a circuit-active drug is judged using only slow tissue endpoints, or where symptom improvement is dismissed as “only symptomatic” despite being the primary disease burden for patients.

Second, safety evaluation has to include host defense logic. Sensory mediators participate in protection and repair in some settings. CGRP-linked programs have been shown to support repair-associated immune behavior after injury in experimental work [116]. Meta-analytic work has also evaluated infection signals with CGRP-pathway preventive therapies, which is directly relevant when considering broadening indications or combining sensory-targeted drugs with immune suppression [247].

Third, stratification matters. The same diagnosis can reflect different circuit states. In atopic dermatitis, clinical heterogeneity maps onto distinct immune endotypes and biomarker patterns, so an itch-dominant neuroimmune-sensitized phenotype will not respond the same way as a broader inflammatory phenotype [290,291]. In asthma, type 2–high and type 2–low endotypes (with differing roles for alarmins such as TSLP and IL-33) support the same point: mechanism-driven targeting needs endotype-aware selection [292,293].

## 8. Conclusions

The recognition of sensory neurons as active immune regulators marks a fundamental shift in the conceptual framework of barrier tissue biology. Instead of operating as separate and independent systems, the nervous and immune systems form an integrated sensory neuroimmune axis that functions as a distributed organ, coordinating host defense, inflammation, and behavior across barrier surfaces [2,16]. This framework brings together previously separate observations in pain, itch, allergy, infection, and tissue repair under one common mechanistic paradigm.

In summary, sensory neuroimmunology has become a key concept in barrier immunity with wide implications for immunology, neuroscience, allergy, infectious disease, oncology, and post-viral syndromes. Viewing sensory neurons as immune sentinels offers a unifying framework for understanding tissue health and disease, laying the groundwork for developing next-generation immunotherapies.

## 9. Future Directions

Despite rapid advances, several critical gaps still exist. One major unresolved area involves how microbial signals influence sensory neuroimmune circuits. Emerging evidence indicates that commensal-derived metabolites, microbial pattern-recognition signals, and epithelial intermediates dynamically adjust neuronal activation thresholds and immune responses, but the molecular mechanisms and circuit-level integration of these signals are still not fully understood [219]. Addressing this gap will be vital for understanding how homeostatic versus pathogenic neuroimmune states are formed and maintained.

Another major challenge is defining human-specific adaptations in sensory neuroimmune circuits. Most current mechanistic knowledge comes from animal models, but increasing human transcriptomic, spatial, and clinical data suggest that neuronal–immune interactions may be uniquely influenced by lifespan, environmental exposures, and complex microbial ecosystems. These factors are especially relevant in systemic and post-infectious conditions like long COVID, where persistent sensory symptoms, immune dysregulation, and barrier dysfunction often occur together. Understanding how sensory neuroimmune circuits contribute to chronic inflammation, unresolved issues, or maladaptive immune memory in humans will be essential for effective clinical application.

In this context, emerging evidence from post-viral syndromes, including long COVID, suggests that persistent neuronal sensitization and impaired resolution of neuroimmune signaling may contribute to sustained inflammation and barrier dysfunction, underscoring the need to mechanistically link sensory neuroimmune circuits to chronic systemic disease.

Therapeutically, the success of anti-CGRP monoclonal antibodies has provided strong proof-of-concept that targeting sensory neuroimmune pathways can deliver significant clinical benefits [65,294]. However, broader neuromodulatory strategies pose important safety concerns. Sensory neurons are vital for host defense, tissue repair, and immune surveillance, and indiscriminate suppression of neuronal signaling may increase infection risk, hinder resolution processes, or cause unintended systemic effects [16]. Future therapeutic approaches must therefore emphasize precision, tissue specificity, and reversibility to maintain protective neuroimmune functions while selectively inhibiting harmful circuits.

Looking ahead, key unanswered questions for the next decade (2025–2035) include the evolutionary origins and conservation of sensory neuroimmune circuits, the extent of human-specific adaptations, the long-term safety of therapeutically targeting neuronal–immune synapses, and how local neuroimmune circuits integrate across organs to affect systemic disease. Addressing these questions will require collaborative efforts combining systems neuroscience, immunology, human translational studies, and advanced spatial and functional technologies.

### Translational Considerations and Limitations

Although several neuro-immune signaling nodes have progressed toward clinical translation, the strength of evidence and safety profile vary substantially across targets. For example, P2X3 receptor antagonists have demonstrated efficacy in refractory or unexplained chronic cough; however, clinical trials consistently report taste disturbance (dysgeusia) due to partial inhibition of related purinergic receptors in gustatory pathways, highlighting a key tolerability limitation. Similarly, therapies targeting the CGRP signaling axis have shown clinical benefit in migraine and are increasingly explored in neuro-immune contexts, yet concerns remain regarding potential cardiovascular and vascular regulatory effects given CGRP’s physiological role in vasodilation. IL-31–directed therapies have provided meaningful symptom relief in chronic pruritus and atopic dermatitis, but their long-term immunomodulatory consequences and disease-modifying capacity remain incompletely defined. In the case of Nav1.8 inhibitors for acute pain, promising preclinical selectivity for nociceptors has not fully translated into consistent clinical efficacy, reflecting challenges in dose optimization, target engagement, and patient heterogeneity. Collectively, these examples underscore a persistent gap between mechanistic findings in experimental systems and reproducible clinical benefit, emphasizing the need for rigorous target validation, improved biomarkers of neuronal–immune circuit engagement, and long-term safety assessment in translational development.

## Figures and Tables

**Table 1:** Landmark Neuroimmune Studies (2022–2025) With Methods, Neuron Types, Effects, and Causal Evidence Labels.

Study (Year)	Method	Neuron Type	Key Effect/Finding	Evidence Label
Deng et al., 2024 [1]	Observational	Sensory neurons (general)	Defined sensory neurons as integral regulators of innate immunity	(associative/observational)
Wang et al., 2024 [2]	Observational	Barrier-associated sensory neurons	Characterized neuroimmune cross-talk at epithelial interfaces	(associative/observational)
Chen et al., 2024 [3]	Observational	TRPV1^+^ nociceptors	Identified TRPV1 as a central node in neuroimmune signaling	(associative/observational)
Liu et al., 2023 [5]	Observational	Cutaneous sensory neurons	Described neuroimmune pathways in atopic and allergic contact dermatitis	(associative/observational)
Feng et al., 2024 [6]	Observational	Cutaneous sensory neurons	Demonstrated neuronal regulation of skin barrier immunity	(associative/observational)
Hanč et al., 2023 [7]	Observational	Nociceptors	Showed nociceptor control of myeloid cell function	(associative/observational)
Erdogan et al., 2025 [9]	Observational	Sensory neurons (general)	Summarized sensory neuron roles in pathogen defense	(associative/observational)
McSwiggin et al., 2025 [14]	Observational (single-cell sequencing)	Airway sensory neurons	Mapped the lung neuroimmune landscape during asthma induction	(associative/observational)
Inclan-Rico et al., 2024 [4]	Functional/targeted neuronal manipulation	Mas-Related G-Protein–Coupled Receptor A3(MrgprA3^+^) pruriceptors	Showed that MrgprA3 neurons drive IL-33–dependent cutaneous immunity	(causal via neuronal manipulation)
Lei et al., 2022 [8]	Experimental (TRPV1-dependent functional assays)	TRPV1^+^ nociceptors	Demonstrated rapid TRPV1-dependent antiviral protein induction after skin injury	(causal via functional perturbation)
Takahashi et al., 2023 [11]	Observational + genetic perturbation	Sensory neurons expressing STAT3	Identified neuronal STAT3 as essential for IL-31R expression and inflammatory itch	(associative/observational)
Crosson et al., 2024 [12]	Observational + cytokine stimulation	Airway sensory neurons	Showed cytokine-mediated transcriptional reprogramming of airway nociceptors in asthma	(associative/observational)
Zhu et al., 2024 [13]	Chemogenetic	TRPV1^+^ gut-associated sensory neurons	Demonstrated that TRPV1^+^ neurons causally regulate Treg cell homeostasis	(causal via chemogenetics)

**Table 2:** Sensory Receptor–Immune Circuits at Barrier Tissues.

Receptor	Primary Stimulus	Neuron Subtype	Immune/Stromal Target	Functional Outcome
TRPV1 (Transient Receptor Potential Ankyrin 1)	Heat (>42 °C), capsaicin, protons (acidic pH), inflammatory mediators	TRPV1^+^ nociceptors (DRG, barrier tissues)	Myeloid cells, macrophages, RORγ^+^ Tregs, fibroblasts, endothelial cells	Neurogenic inflammation, pain hypersensitivity, itch, fibrotic remodeling, immune modulation
TRPA1	Reactive electrophiles, oxidative stress, environmental irritants	TRPA1^+^ polymodal nociceptors	Immune cells (macrophages, mast cells), epithelial cells	Inflammatory amplification, oxidative stress sensing, pain and pruritus signaling
Nav1.8 (SCN10A)	Membrane depolarization (TTX-resistant sodium conductance)	Nav1.8^+^ primary sensory neurons	Antigen-presenting cells, cytokine-producing immune cells	Sustained action potential firing, chronic pain facilitation, cytokine modulation
Nav1.7–1.9	Voltage-gated sodium activation	Nociceptive sensory neurons	Indirect immune modulation via neuronal hyperexcitability	Paroxysmal pain and itch phenotypes
MrgprA3	Chloroquine, pruritogens, immune checkpoint–associated cytokine signaling	MrgprA3^+^ pruriceptive DRG neurons	Macrophages, cDC2, IL-17^+^ γδ T cells	Chronic itch, IL-17/23 axis modulation, cytokine reprogramming, neuroimmune feedback loops
Histamine H1–TRPV1 axis	Histamine	TRPV1^+^/H1R^+^ pruriceptive neurons	Local immune cells	Acute histaminergic itch
PAR2–TRPV1/TRPA1 axis	Proteases	Polymodal sensory neurons	Immune and epithelial cells	Non-histaminergic itch, chronic pruritus

**Table 3:** Summary of key mediators, their neuronal sources and corresponding immune receptors, the primary immune effects of each pathway, and associated protective and pathological roles.

Mediator	Neuronal Source	Immune Receptor	Primary Immune Effect	Protective Role	Pathological Role	References
CGRP	TRPV1^+^ nociceptors	RAMP1/CALCRL (on ILC2s, macrophages)	Suppresses IL-13; promotes IL10/resolution	Mucus production; anti-helminth balance	Chronic itch in AD; neurogenic inflammation	[13,123]
Substance P	Peptidergic C-fibers	NK1R; MRGPRX2/B2 (mast cells)	Mast degranulation; cytokine release (TNF-α)	Rapid defense against bacteria	Psoriasis amplification; pain hypersensitivity	[2]
VIP	Cholinergic/enteric neurons	VPAC1/2 ILC2s/Th2) (on	Enhances IL-5; bronchodilation	Type 2 immunity in helminths	Asthma exacerbation	[124]
IL-33	Epithelial/immune cells	ST2 (on neurons)	Neuronal sensitization; itch induction	Alarm response to damage	Chronic allergic itch	[125]
IL-31	Th2 cells	IL-31RA (on pruriceptors)	Direct itch signaling	Parasite expulsion	Atopic dermatitis pruritus	[123]
TSLP	Keratinocytes	TSLPR (on neurons)	Activates TRPA1^+^ neurons	Barrier alert	Feed-forward AD loops	[2]

**Table 4:** Current and Emerging Therapeutics Targeting Sensory Neuroimmune Circuits.

Target	Drug/Class	Mechanism	Approved Indications	Pipeline Indications	Key Trial/Evidence (2024–2025)	Limitations
CGRP/RAMP1	Erenumab, fremanezumab, galcanezumab, eptinezumab; gepants (e.g., atogepant)	Block CGRP or CGRP receptor	Migraine prevention	Select itch/barrier indications under study	APPRAISE trial; Phase 3 data in migraine prevention [243,244,245,246]	Context-dependent immune/repair roles; infection/host defense signal monitoring [116,145]
Nav1.8	Suzetrigine (JOURNAVX)	Peripheral voltage-gated Na^+^ channel inhibition	Acute pain (FDA 2025) [247]	Chronic pain, possibly itch	FDA approval and label (2025) [247,248,249]	Selectivity and longer-term safety for chronic use; off-target risks
Nav1.7	Selective blockers (multiple classes)	Voltage-gated Na^+^ channel inhibition	None	Chronic pain, itch	Ongoing clinical programs; efficacy varies by modality	Cardiac/CNS off-target effects; translation from genetics to pharmacology [248,249]
IL-31/IL-31RA	Nemolizumab (NEMLUVIO)	IL-31RA blockade	Atopic dermatitis (FDA 2024) [250]	Prurigo nodularis	Label + pivotal evidence summarized in prescribing info [250]	Injection-site reactions; may require upstream anti-inflammatory control in some endotypes [249]
JAK pathway	Abrocitinib; other JAK inhibitors	Cytokine signal transduction inhibition	Atopic dermatitis; IBD (selected agents)	Broader pruritic/inflammatory conditions	Rapid itch time-course data; approved indications [251,252]	Serious infection/thrombotic/cardiovascular risks in selected populations
P2X3	Gefapixant; camlipixant (in trials)	Blocks ATP-mediated sensory activation	None (varies by region/program)	Refractory chronic cough	COUGH-1/COUGH-2; SOOTHE trial [174,253]	Taste disturbance; heterogeneous responder biology
IL-33/ST2	Astegolimab, etokimab (examples)	IL-33 or ST2 blockade	None	Asthma, AD	Phase II/III development	Broad immune modulation risk; host defense constraints
MRGPRX2	Small-molecule antagonists (e.g., EVO756 program)	Reduces non-IgE mast cell activation	None	Urticaria-like disease, allergic itch	Phase 1 target engagement reports [254]	Species and receptor pharmacology gaps; program attrition in adjacent targets [255]
BTK	Brain-penetrant BTK inhibitors	Modulates microglia/B-cell–linked neuroinflammation	None	Progressive MS; proposed symptom-linked neuroinflammation	NEJM MS trial; mechanistic microglia work [256,257]	Systemic safety constraints; endpoint specificity beyond symptom scores [258]
Gene/circuit silencing	Optogenetic-inspired or gene-enabled neuromodulation	Targeted reduction in afferent activity	None	Chronic pain (selected settings)	Preclinical proof; translational reviews [259,260]	Delivery, reversibility, long-term safety; regulatory pathway complexity

**Table 5:** Comparative Anatomy of Sensory Neuroimmune Circuits Across Barrier Tissues.

Tissue	Dominant Neuron Subtypes	Major Neuropeptides	Key Immune Partners	Primary Reflex	Prototypical Disease	Therapeutic Successes
Skin	TRPV1^+^/TRPA1^+^ C-fibers; MrgprA3^+^ pruriceptors	CGRP, Substance P	Mast cells, ILC2s, γδ T cells, macrophages	Itch-scratch cycle	Atopic dermatitis, psoriasis	Anti-IL-31 (nemolizumab); anti-CGRP repurposing
Lung	Vagal/TRPV1^+^ afferents	VIP, CGRP	ILC2s, eosinophils, macrophages	Cough; bronchoconstriction	Asthma, allergic airway inflammation	Anti-TSLP (tezepelumab); P2X3 antagonists (gefpixant trials)
Gut	Enteric/TRPV1^+^ nociceptors	CGRP, VIP, NMU	ILC2s/3s, Tregs, muscularis macrophages	Peristalsis; mucus secretion	IBD, helminth infection	Emerging: NMU analogs; Treg-modulating via TRPV1

## Data Availability

No new data were created or analyzed in this study. Data sharing is not applicable.

## Abbreviations

AD	atopic dermatitis
CGRP	calcitonin gene-related peptide
DRG	dorsal root ganglion
GPCR	G protein–coupled receptor
ILC	innate lymphoid cell
LPS	lipopolysaccharide
MRGPR	Mas-related G protein–coupled receptor
TSLP	thymic stromal lymphopoietin
TRP	transient receptor potential
VIP	vasoactive intestinal peptide
